# Navigating the contemporary landscape of food waste management in developing countries: A comprehensive overview and prospective analysis

**DOI:** 10.1016/j.heliyon.2024.e33218

**Published:** 2024-06-19

**Authors:** Tawfikur Rahman, Nibedita deb, Md Zahangir Alam, Md Moniruzzaman, Md Shohidullah Miah, Mohammad Abu Horaira, Reashad Kamal

**Affiliations:** a Department of Electrical and Electronics Engineering, International University of Business Agriculture and Technology, Uttara, Dhaka, 1230, Bangladesh; b College of Agricultural Sciences, International University of Business Agriculture and Technology, Uttara, Dhaka, 1230, Bangladesh; c Bioenvironmental Engineering Research Center (BERC), Department of Chemical Engineering and Sustainability, Faculty of Engineering, International Islamic University Malaysia, 50728, Kuala Lumpur, Malaysia; d College of Tourism and Hospitality Management (CTHM), International University of Business Agriculture and Technology, Uttara, Dhaka, 1230, Bangladesh; e Department of Pharmacy, Daffodil International University, Dhaka, Bangladesh

**Keywords:** Processing and recycling, Food waste, Policy and regulation, Sustainable development, Management system

## Abstract

This study employs a comparative analysis method to examine variations in food waste (FW) generation between developed and developing nations, focusing on income levels, population growth rates, and community engagement in waste management. Quantitative data from Taiwan, Malaysia, and Bangladesh are comprehensively analyzed using regression analysis and descriptive statistics. Results indicate that Taiwan, with its stringent regulatory frameworks and advanced recycling technologies, generates significantly less FW per capita compared to Malaysia and Bangladesh. Malaysia shows moderate levels of FW reduction efforts, supported by varying degrees of community participation, whereas Bangladesh faces challenges with both regulatory enforcement and technological adoption. The study proposes an integrative waste management model emphasizing regulatory compliance rates, community participation metrics, and technology diffusion indices to effectively address FW challenges. These findings underscore the importance of tailored waste management strategies aligned with economic and demographic contexts of developing nations. Policymakers and waste management practitioners can leverage these insights to establish targeted FW reduction goals and enhance recycling initiatives. The research highlights the urgency of integrated waste management approaches to mitigate environmental and public health risks associated with FW mismanagement, advocating for evidence-based policies supported by robust quantitative analysis.

## Graphical abstract

**Figure:**
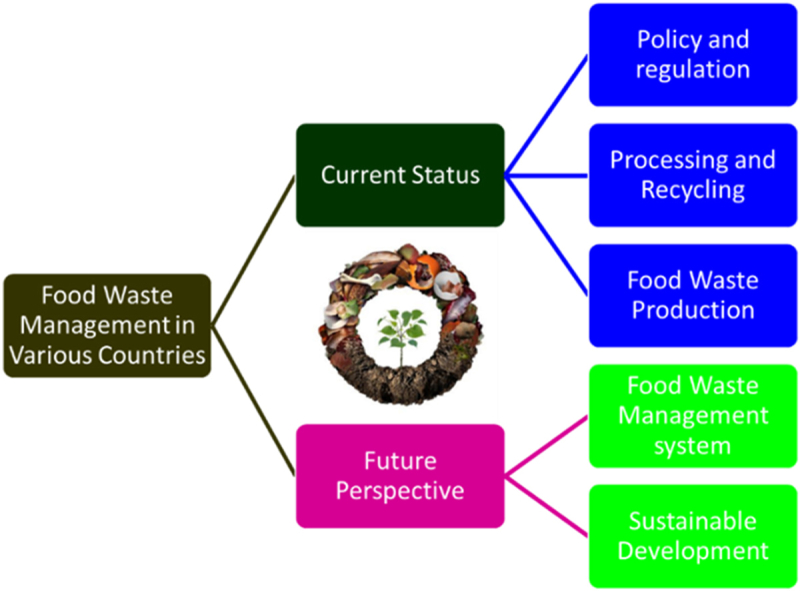

## 1 Introduction

Food waste (FW) in different country primarily generated from households and food service establishments. The observation that fruits and vegetables, cooked food, and bread are the most frequently wasted food items may not necessarily be specific to regions with cold climates. Food wastage patterns can vary based on various factors including cultural preferences, dietary habits, availability of certain foods, and socio-economic conditions rather than solely on climate. Therefore, while climate can influence food production and availability, the types of foods most frequently wasted are often influenced by a complex interplay of cultural, economic, and behavioral factors rather than solely climate conditions. It also identified several reasons for food waste in several country, including overproduction, a lack of awareness and knowledge about food waste management, and cultural practices discouraging leftover food consumption [1]. However, the classification of food waste sources (FW sources) refers to categorize the various stages or phases in the food supply chain where food waste occurs [2]. This classification helps in identifying the different sources of food waste and in developing strategies to reduce food waste. The classification of food waste sources can vary depending on the context or country. For example, the European Commission categorizes FW origins into three groups: food losses during production, inescapable waste generated during consumption, and preventable waste during consumption [3]. On the other hand, it separates food waste into five generation sources depending on the stages of supply chain: farming production, handing products after harvesting and storing, processing, and supply along with consumption. The classification of food waste sources assists researchers and policymakers in pinpointing the stages where the most significant food waste occurs and in developing targeted interventions to reduce food waste at those stages. These losses may occur due to various factors, such as inadequate storage, transportation, processing, and packaging methods, natural disasters, pests, and diseases, among others [4]. Food losses can occur at different levels in chain of the supply, comprising on-farm losses in harvesting, post-harvest losses during handling, storage, and transportation, and retail losses at supermarkets and restaurants. The extent and causes of food losses vary depending on the type of food and the region [5]. In addition, food losses not only result in significant economic losses for farmers, food producers, and retailers but also have significant environmental and social implications. Waste Food contributes to greenhouse gas emissions, and exerts negative impact on land and water, and exacerbates food insecurity and hunger in many regions. Unavoidable food wastes are produced due to reasons beyond human control, such as bones, shells, peels, and other inedible parts of food. These types of waste are spawned during processing of food, preparation along with the consumption and cannot be consumed or repurposed [6].

Unavoidable food waste can also include food that becomes spoiled or contaminated, posing a health risk if consumed. This type of waste cannot be avoided, as it is essential to protect human health and safety. Although unavoidable food waste cannot be completely eliminated, efforts can be made to reduce its impact. For example, inedible food waste can be used for composting, enriching soil, and reducing the need for synthetic fertilizers. Additionally, new technologies can be developed to convert inedible food waste into useable resources, such as biogas for energy production. Overall, while unavoidable food waste cannot be entirely eliminated, we can find ways to minimize its impact on the environment and society. Avoidable food waste refers to food that is discarded even though it is still edible and could be consumed [7]. This type of waste often results from inefficient food management practices, such as overproduction, over-purchasing, or improper storage, handling, and preparation. Examples of avoidable food waste include fruits and vegetables not sold due to cosmetic imperfections, expired or spoiled food not used before the expiration date, and leftovers discarded instead of being stored or repurposed for another meal. Avoidable food waste has significant social, environmental, and economic consequences. This leads to the wastage of precious resources like water, energy, and land employed in food production, while also producing greenhouse gas emissions that contribute to the issue of climate change [8]. Additionally, it exacerbates food insecurity and hunger, as food that could have been used to feed people is discarded. To reduce avoidable food waste, individuals and organizations can take several measures, such as carefully planning meals to avoid over-purchasing, donating surplus food to food banks or shelters, using leftovers creatively to make new meals, and composting food scraps. Such practices not only reduce food waste but also save money and conserve resources, promoting sustainability and environmental stewardship. This study focuses on the issue of FW, which can occur during the consumption phase and can be either avoidable or unavoidable. The definition of food waste used in this study is based on the definitions put forth by Brian et al. [9], stating that FW is the refers to the impairment of food in food chain's final stage, specifically in retailing and consumption levels, because of the attitude of vendors and customers. It includes food that is still suitable for human intake but is cast-off either beforehand or afterward of spoiling, due to slackness or a cognizant choice of throwing [[7], [8], [9]].

In unindustrialized nations, the primary source of food waste is households. These countries have large and diverse populations, which result in a substantial volume of food waste. This issue is of concern for both economic and social reasons, as a significant portion of the population lives below the poverty line. In urban settings, households contribute significantly to food waste due to factors like excessive purchasing, food spoilage, and improper storage [10]. Food waste is also a notable problem in restaurants and other food service establishments where considerable amounts of food are prepared and served daily. In rural areas, food waste primarily occurs during agricultural and processing stages. Farmers often discard produce considered unsuitable for sale due to cosmetic imperfections, while food processing units generate waste from by-products such as peels, skins, and seeds. This substantial amount of food waste carries noteworthy environmental and economic consequences [11]. It results in the inefficient use of valuable resources like water and land dedicated to food production and generates greenhouse gas emissions that contribute to climate change. Efforts are underway in the country to tackle the food waste issue, including initiatives promoting sustainable food consumption habits and reducing waste at the household and community levels [11,12].

Based on the information provided, a developing country since its GNI per capita per year is below the threshold of US$11,905 set by the International Statistical Institute [12]. This categorizes of country with a low to middle-income economy. It's important to note that classifying a country as "developing" or "developed" based solely on its GNI can be a simplistic approach, as other factors can influence a country's economic and social development. These factors encompass access to education, healthcare, and technology, as well as political stability, infrastructure, and natural resources. Furthermore, while GNI can serve as an indicator of a country's economic progress, it may not necessarily reflect wealth distribution within a country or the standard of living of its citizens. Hence, it's essential to explore alternative indicators, like the Human Development Index (HDI), which factors in elements such as life expectancy, education, and income, to obtain a more holistic perspective on a nation's progress [12].

Developing nations encounter more substantial hurdles when it comes to food waste management in contrast to developed nations. Inadequate separation of food waste from Municipal Solid Waste (MSW) results in heightened greenhouse gas emissions at landfill sites, posing an environmental issue. Isabelle Denis [13], from the FAO Liaison Office in Brussels has suggested that food waste can generate greenhouse gas emissions, contributing to climate change. Therefore, countries need to recognize and implement essential food waste management practices such as prevention, recycling, and disposal. A comprehensive overview of food waste management can assist in addressing food waste issues. An assessment is accomplished in this article on the present status of food waste production, strategies, disposal methods that are followed in various countries. This assessment is helpful for undertaking necessary steps in countries of developing stage for managing waste of food in near future.

## 2 The present situation of FW management in developing and underdeveloped nations

The existing state of FW management varies widely in developing and underdeveloped countries. In many developing nations, FW is still predisposed of in landfills alongside other municipal solid waste without segregation or treatment, resulting in significant environmental issues such as greenhouse gas emissions, groundwater contamination, and soil pollution. Additionally, due to poor infrastructure and inadequate waste collection services, food waste often accumulates in public spaces, leading to health hazards and unpleasant odors [14]. However, some developing countries have taken effective steps to manage FW. For instance, countries like South Korea, Japan, and Singapore segregate FW at its source, and the collected waste is converted into energy or compost through various treatment methods. In other developing nations, such as Brazil and India, innovative approaches like community composting and animal feed production are being implemented to reduce food waste. In underdeveloped countries, food waste management is often nonexistent or minimal due to poverty, lack of resources, and inadequate infrastructure. Food waste is either fed to livestock or left to decompose, causing environmental and public health problems. There is a significant need for developing and underdeveloped countries to prioritize effective food waste management by implementing sustainable and cost-effective solutions [15].

### 2.1 Production of FW

The generation of FW is a noteworthy worldwide issue, and it exhibits variations among different countries. According to reports from the Food and Agriculture Organization (FAO), roughly one-third of the food produced globally goes to waste annually, equating to around 1.3 billion tons. Wasted food is mainly generated in households in developed countries as well as from food service industry. In contrast, in developing countries, it occurs all over the food resource chain due to inadequate transportation and storage facilities, insufficient processing technology, and limited market access [16]. Food waste can also result from consumer behavior, such as purchasing excessive food, over-preparing meals, and discarding edible food due to expiration dates or aesthetic imperfections. Additionally, food waste occurs in supermarkets and other retailers when products do not meet strict cosmetic standards and are discarded. The production of FW has substantial cost-effective, community, and eco-friendly influences, as well as the loss of valuable resources such as land, water, and energy, increased greenhouse gas emissions, and food insecurity among vulnerable populations. Therefore, reducing FW should be a significance for all stakeholders in the food supply chain [17].

The generation of FW can be quantified by considering the total weight of FW produced annually, typically measured in tons per year, and on a per capita basis, often expressed in kilograms per year or kilograms per day for individual consumers. Table 1 shown that the variations in food waste generation across Developed and Developing Nations. In Europe and North America, the per capita FW generated by consumers falls within the range of 95–115 kg per year (kg/year), as reported by Gustavsson et al., [4]. In contrast, sub-Saharan Africa and South/Southeast Asia exhibit a much higher per capita FW generation rate, ranging from 6 to 11 kg/year, as indicated in the same study. A more recent study by Sinha et al. [15], reveals a significant disparity in per capita FW generation between developed and developing countries. In developed countries, the average per capita FW generation is estimated at 107 kg/year, while in developing countries, it is substantially lower at 56 kg/year. These findings underscore the clear distinction in FW generation patterns between regions with higher living standards and those with lower living standards, where higher living standards are associated with greater FW generation, as discussed by Brian et al., [9]. This disparity can be attributed to the fact that higher living standards in developed countries are linked to elevated expectations for food quality and aesthetics among consumers. Consequently, there is a greater demand for high-quality food products, necessitating more ingredients and resources in the production process, ultimately leading to larger quantities of FW generation. Additionally, consumer behavior can influence the amount of FW generated by retailers, as unsold or expired food products are often discarded rather than donated to food charities or shelters [18].

**Table 1:** Variations in food waste generation across Developed and Developing Nations.

Name of Countries	Population (1950–2022)	Total FW (tonne/year)	FW per capita (kg/day)	FW **(MSW)** (%)	GNI (US$)	Reference
**Developed Countries**						
Norway	5,434,319	453,650	0.21944	55 %	82,840	Orr et al. [16]
Denmark	5,882,261	790,502	0.225	N/A	66,720	Aschemann et al. [17]
Australia	26,124,814	2,261,061	0.283	40 %	55,660	Cook et al. [18]
Sweden	10,549,347	1,915,460	0.225	N/A	61,090	Weber et al. [19]
Singapore	5,975,689	796,000	0.222	10 %	102,450	Kua [20]
United States	333,287,557	60,849,145	0.1638	30 %	70,480	Gallo [21]
Germany	83,369,843	12,257,998	0.2083	59 %	59,630	Orr et al. [16]
South Korea	51,815,810	6,241,500	0.1972	2.60 %	47,770	Yadav et al. [22]
United Kingdom	67,508,936	14,257,000	0.2138		49,420	Facchini et al. [23]
**Developing Countries**						
Brazil	215,313,498	33,489,000	0.167	N/A	15,600	Moraes et al. [24]
Turkey	85,279,553	12,375,000	0.2583	N/A	30,290	Tozlu et al. [25]
Malaysia	33,938,221	5,477,263	0.25278	N/A	28,150	Lim et al. [26]
China	1,411,750,000	195,000,000	0.178	N/A	19,160	Cheng et al. [27]
Thailand	71,697,030	9,312,788	0.2194	N/A	18,120	Walter et al. [28]
India	1,417,173,173	71,952,838	0.1389	40 %	7130	Sahoo et al. [29]
Bangladesh	171,186,372	10.62 million	0.18056	5.50 %	6840	Datta et al. [30]
South Africa	59,893,885	10 million	0.1111	N/A	14,340	Kurniawan et al. [31]
Indonesia	275,501,339	23 million	0.21389	N/A	12,680	Prihadyanti,[23]

Societies characterized by lower living standards typically have less stringent demands for food quality, leading to lower per capita food waste (FW) generation [16]. However, it's important to note that despite these lower individual waste generation rates for developing nation, the total amount of FW is not significantly lower than that of developed nations. The combined impact of population growth as well as ever-increasing financial challenges in developing nations are some key reasons for this [19]. As per FAO report of 2023, the global annual FW generation amounts to approximately 60,849,145 tons per year [21]. Interestingly, this study found no significant disparity between developed countries, where the annual FW generation was estimated at 804 million tons, and developing countries, where it stood at 768.6 million tons. This apparent balance can be attributed to the interplay of several factors [20]. Firstly, as per a recently published report from the United Nations, the current global population of 7.6 billion is anticipated to rise to 8.6 billion by 2030, 9.8 billion by 2050, and a staggering 11.2 billion by 2100. This growth trajectory remains consistent, with approximately 83 million individuals being added to the world's population each year, even if fertility rates decline [21]. While the individual generation rates of FW in developing countries are lower due to reduced food demand and consumption standards, the higher overall populations of 137 developing nations, in contrast to 49 developed nations, result in a total FW generation that is nearly equivalent to that of developed countries [[24], [25], [26], [27], [28], [29], [30], [31]]. This phenomenon emphasizes the significance of considering both per capita and total FW generation within a global context, while taking into account the intricate interplay between population size, economic development, and waste generation patterns [28].

FW information for various countries indicates that FW is carefully related to factors such as population growth and the Gross National Income (GNI) index, this study primarily analyzes the collected data with a focus on the GNI index as a key determinant by Cheng et al., [27]. The summary of the FW generation landscape is presented in Table 1. In general, FW in developing countries tends to account for approximately 50 %–55 % of the total municipal solid waste (MSW) generated, a finding consistent with the study conducted by Ref. [23]. The data in Table 1 highlights specific examples, indicating that in countries such as Malaysia, Brazil, India, Mexico FW constitutes 55 %, 54.9 %, 51 %, 52 % of MSW, respectively. This significant organic fraction within the waste stream implies that composting is a highly viable method for FW treatment in developing nations due to the rich source of organic materials. Furthermore, there is a concerning trend in the global urban context, with projections suggesting that urban FW will surge by 44 % between 2005 and 2025, as indicated by Datta et al., [30]. This increase is particularly pronounced in Asian nations due to their rapid economic development. It is estimated that in Asia, FW generation can varies within 278 million tons to 416 million tons, potentially contributing to global anthropogenic emissions within the range of 8 %–10 % by Lim et al., [26]. In essence, this data underscores the significance of considering economic development, population growth, and regional variations when assessing FW generation patterns. It highlights the need for targeted strategies and policies to address the mounting challenge of FW in rapidly developing regions, particularly in Asia, and the potential environmental consequences associated with this increase [25].

**Fig. 1:**
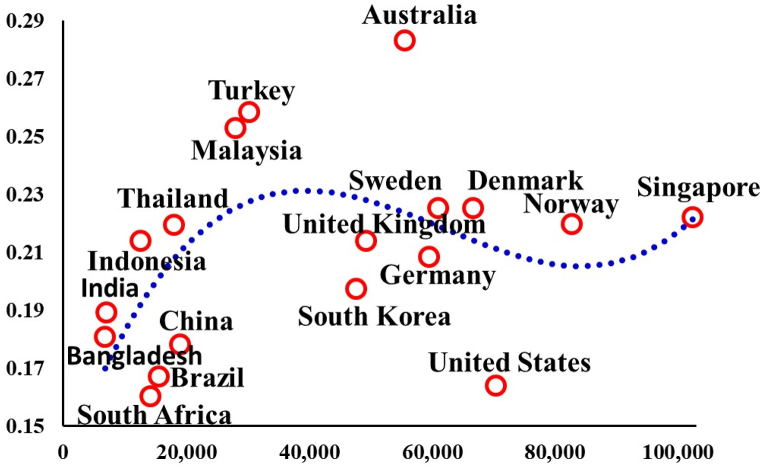
GNI per capita from 1990 to 2021.

Fig. 1 depicts the connection between Gross National Income (GNI) per capita and per capita food waste (FW) in various countries. This visual representation unveils a clear link between income levels, represented by GNI per capita, and the rate at which food waste is produced. When examining developed nations, the pattern of food waste generation can be divided into two distinct categories. In the first group, characterized by countries with GNIs exceeding US$42,000, FW generation exhibits a gradual decline as income levels rise. This reduction in FW generation is attributed to the implementation of "zero waste" policies in these developed nations, such as Australia, Singapore, Norway, the United States, Germany and Sweden, as reported by Cook et al., [18]; Kua, [20]; Orr et al., [16]; Gallo, [21]; Orr et al., [16]; Weber et al., [19]. The main beliefs of "zero waste" initiatives pertaining to FW emphasis on rising digression rates and minimizing waste generation throughout food making developments. In nations where "zero waste" policies are effectively adopted, waste generation, including FW, approaches minimal levels, resulting in a gradual decrease in FW generation per capita, as highlighted. Conversely, the second group of developed countries, characterized by GNIs below US$102,450, experiences a different trend in FW generation. In these nations, "zero waste" policies have not been widely adopted, leading to a lower diversion rate for FW and higher levels of virgin waste generation during food production processes [[18], [19], [20], [21]]. Consequently, per capita FW generation remains high in this group due to the persistence of suboptimal waste management practices. Table 1 demonstrates a notable association between income levels (GNI per capita) and FW generation rates in various countries. It distinguishes between developed countries with different approaches to waste management, showcasing how "zero waste" policies can substantially reduce FW generation per capita, while others continues to grapple with higher levels of FW due to less effective waste diversion strategies. This analysis underscores the importance of policy interventions and waste management practices in shaping FW generation trends in different regions [[23], [24], [25], [26], [27], [28], [29], [30], [31]]. In contrast, the dynamics of FW generation in developing nations appear to follow a different pattern, where wealthier nations (characterized by high Gross National Income or GNI per capita) tend to generate higher levels of FW per capita [30]. Several key factors influence the FW production rates in developing nations, including population growing and the pace of expansion, both of which are intricately linked to income stages, as documented in the study by Sahoo et al., [29]. To illustrate this trend, consider the example of Costa Rica, where a small-scale population is associated with the maximum FW production rate of 0.25 kg per day (kg/day).

In contrast, India and South Africa, characterized by its large-scale population, records the lowest FW generation rate at 0.14 and 0.11 kg/day. This phenomenon is indicative of the influence of population size and economic development on FW generation trends within developing countries. Furthermore, Table 1 offers valuable insights into the overall FW generation landscape in developing nations. Among the total FW generation amounting to 630 million tons across all developing states, India and China emerge as the primary providers, accounting for 195 and 72 million tons, respectively. These Fig. 1 underscore the significant role played by these populous countries in the aggregate FW generation within the developing world. The relationship between wealth (as indicated by GNI per capita) and FW production in developing states differs from that observed in developed countries [27]. Here, the trend suggests that higher-income nations within the developing world tend to generate more FW per capita. This trend is shaped by influences such as population progress, urbanization rates, and commercial growth levels. The data in Table 1 emphasizes the substantial contributions of China and India to the total FW generation in developing states, highlighting the importance of considering these populous nations in any efforts to address FW challenges on a global scale.

In developing country, FW generation is a significant concern that has adverse influences on public health, the environment, and the low-cost. According to a study conducted by the World Bank, the country generates approximately 10.62 million of FW per year, with the majority of the waste being produced in urban areas [30]. The key sources of FW in developing country are households, food markets, and the food service industry. In households, food waste often results from over-preparation, improper storage, and food spoilage due to inadequate refrigeration and poor hygiene practices [31]. In food markets, significant amounts of fruits and vegetables are discarded due to poor storage and handling practices, while in the food service industry, FW is generated during preparation, serving, and post-consumption. Food waste in Bangladesh poses a severe threat to public health, as it creates breeding grounds for insects and rodents, leading to the spread of diseases. Additionally, food waste generates significant greenhouse gas emissions, contributing to climate change [30]. The disposal of food waste also creates a financial burden for local governments, requiring additional resources for waste management. To address this issue, Bangladesh needs to develop effective food waste management policies and strategies that include measures to reduce food waste at the source, proper segregation and collection, treatment and disposal options, and public awareness campaigns to promote responsible consumption practices. Innovative approaches such as composting, animal feed production, and biogas generation can also provide sustainable solutions to food waste management in developing country [[25], [26], [27], [28], [29], [30], [31]].

### 2.2 Food waste recycling activities

**Fig. 2:**
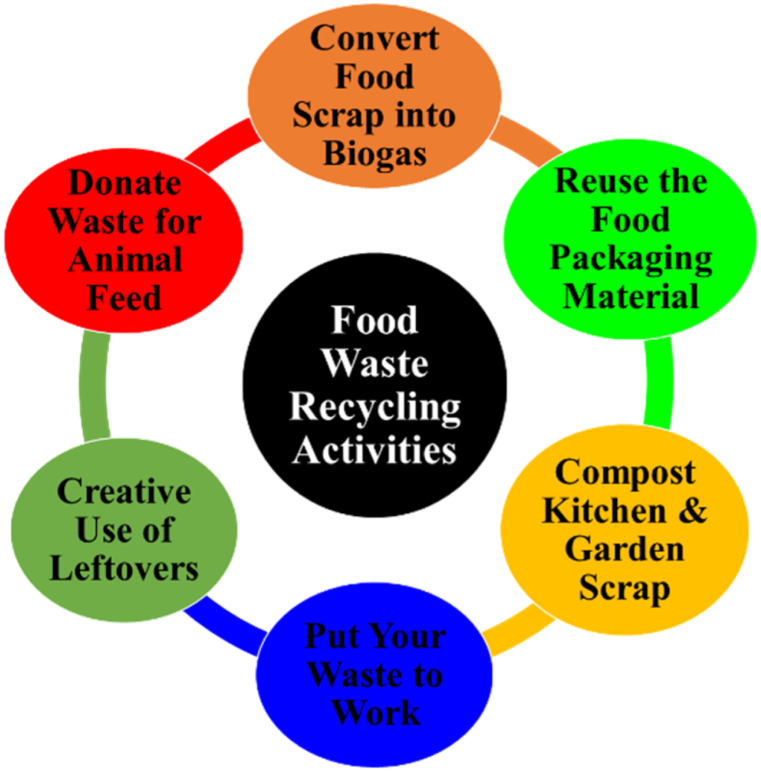
Six environment-Friendly ways to food waste recycling activities.

The current status of food waste (FW) recycling activities in developing and underdeveloped countries presents a range of challenges and opportunities that vary significantly across regions. This paper elaborates on key points related to FW recycling in these countries, as shown in Fig. 2.

Developing and underdeveloped countries often face challenges with inadequate waste management infrastructure, including insufficient facilities for sorting, collecting, and processing FW. Many regions lack dedicated composting or anaerobic digestion facilities necessary for converting organic waste into valuable resources like compost or biogas [26]. Additionally, some developing countries lack comprehensive policies and regulations addressing FW recycling and management. The absence of clear guidelines and incentives can impede efforts to promote recycling practices and develop recycling infrastructure. Informal sectors often play a significant role in FW recycling, with waste pickers and scavengers collecting recyclables, including FW, from landfills and streets. However, this informal sector is typically unregulated and lacks safety measures. Effective FW recycling often begins at the source, with households and businesses separating organic waste from non-organic materials [27]. Achieving high levels of source separation can be challenging in countries with limited public awareness and education about recycling. Economic limitations can restrict investments in recycling infrastructure and technology, making it challenging to establish efficient recycling processes. Low budgets for waste management may lead to inadequate waste collection and disposal systems, exacerbating the issue.

Promoting community engagement and raising awareness about the benefits of FW recycling is crucial. Developing educational programs can help change attitudes toward waste, encourage responsible disposal, and boost participation in recycling initiatives [26]. Despite these challenges, there is significant potential for improvement in FW recycling in these countries. Innovative approaches, such as community-based recycling initiatives, partnerships with non-governmental organizations (NGOs), and technology transfer, can help jumpstart recycling efforts. Some developing and underdeveloped countries have successfully implemented FW recycling programs and policies, offering valuable lessons for others. Examining successful models from countries like China, India, and Brazil can provide insights into effective waste management and recycling strategies [24,27,29]. For example, Malaysia implemented household waste separation legislation in 2023, requiring separate bins for organic and recyclable waste [27]. Thailand's government has promoted FW separation at the source in select cities, aligning with their "3Rs" implementation aimed at increasing organic waste utilization by 50 % before 2026 [29]. Thailand's government has collaborated with NGOs to facilitate composting programs by providing free organic waste bins nationwide [29].

However, challenges persist in FW recycling systems, including incomplete FW management infrastructure, limited markets for FW products, and inadequate financial incentives. In India, despite substantial FW volume, recycling activities remain limited, with organic waste primarily disposed of in dumpsites [30]. China also faces challenges with a weak recycling system characterized by inadequate collection infrastructure and treatment facilities. While some large Chinese cities have implemented source separation and home collection, hotels and restaurants often manage FW using biological processes [28]. Only a few FW treatment facilities operate in China, with significant FW separation and treatment occurring in major cities [28]. The status of FW recycling activities in developing countries reveals several key aspects. Notably, developing countries have a significant informal recycling sector, with waste pickers and scavengers playing a crucial role in collecting recyclable materials, including FW, from landfills and streets [31]. However, this informal sector is often unregulated and faces safety and health risks.

Bangladesh lacked comprehensive national policies or regulations specifically targeting FW recycling at that time, contributing to the absence of clear guidelines and incentives for formalized recycling practices [30,31]. Achieving effective source separation of waste, including FW, posed a challenge due to limited public awareness and education on recycling. Rapid urbanization and population density in cities exacerbated waste management issues. Economic constraints limited investments in recycling infrastructure, affecting the establishment of efficient recycling processes. Non-governmental organizations (NGOs) and international organizations have been involved in promoting sustainable waste management practices in developing countries. While there is potential for improvement in FW recycling, government initiatives and developments in this area may have evolved since 2023 [31]. The recycling of FW in developing countries faces a multitude of challenges, ranging from policy gaps to infrastructure limitations and insufficient incentives. Collaborative efforts involving governments, NGOs, businesses, and communities are essential to overcoming these challenges and promoting more efficient FW recycling practices in these regions [32,33]. Table 2 shows the systematizing FW recycling activities in different countries along with corresponding data could be useful for analysis.

**Table 2:** Food waste recycling activities in different countries.

Country	FW Recycling Initiatives	Key Challenges	Successes and Innovations
**India**	Limited recycling activities; organic waste primarily disposed in dumpsites	Lack of infrastructure; low public awareness	Efforts to promote FW composting; initiatives by NGOs
China	Weak recycling system; some source separation in major cities	Inadequate collection infrastructure; limited treatment facilities	FW treatment facilities in major cities; biological processes in hotels/restaurants
Brazil	Informal recycling sector; incomplete legislative frameworks	Policy gaps; insufficient education programs	Collaborations with NGOs for composting programs
Mexico	Challenges with recycling systems due to legislative gaps	Lack of comprehensive educational curriculums	Initiatives to improve FW separation and collection
Malaysia	Household waste separation legislation implemented in 2023	Financial constraints; incomplete infrastructure	Separate bins for organic and recyclable waste
Thailand	FW separation promoted in select cities; "3Rs" implementation	Incomplete FW management infrastructure	Collaboration with NGOs for nationwide composting programs
Bangladesh	Lack of comprehensive national policies for FW recycling	Limited public awareness and education	Potential for development with international assistance
United States	Diverse recycling initiatives; advanced composting facilities	High waste generation; challenges in urban areas	Advanced technologies for FW processing and conversion

This Table 2 provides a structured overview of FW recycling activities, challenges, and successes across different countries, allowing for comparative analysis and insights into global FW recycling practices.

### 2.3 Current food waste treatments

**Fig. 3:**
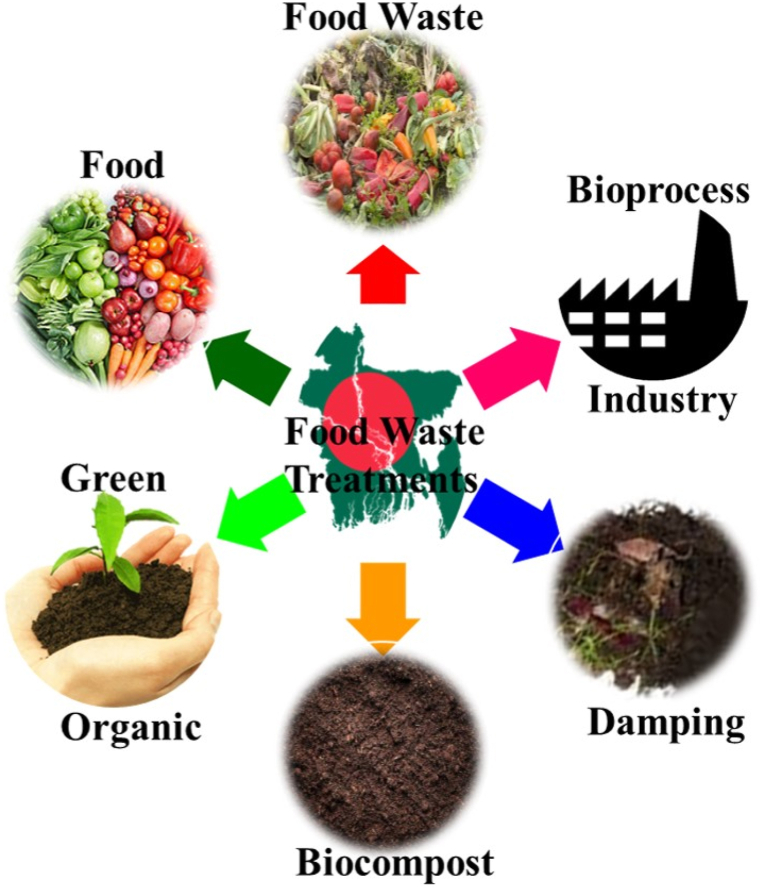
Common food waste treatment process.

Food waste handling procedures in developing as well as underdeveloped countries vary based on available resources, infrastructure, and local practices. There are some common food waste treatment methods widely employed in developing nations, namely animal foods, fertilizer production, anaerobic assimilation, incineration, landfill disposal [32]. Among these methods, illegal open dumps and landfills have been extensively practiced in this region as shown in Fig. 3. Dumping and landfills are accountable for 90 % waste disposal. On the other hand, composting is responsible for 1 %–6 % of disposal process across different areas. In contrast, anaerobic digestion contributes under 0.6 %, and animal feeding, incineration are seldom practiced for managing food waste in these regions. This information highlights the dominance of landfills and open dumps as the default method for food waste disposal in developing countries, despite their environmental and health challenges. It also underscores the need for increased efforts and investment in sustainable and environmentally friendly FW treatment alternatives, such as improved composting techniques and anaerobic digestion systems, to mitigate the negative impacts associated with the prevalent disposal methods [33]. Landfill disposal remains a prevalent method for handling food remaining in many developing and underdeveloped countries. Food waste is typically mixed with other waste streams and buried in landfills, contributing to environmental concerns such as methane emissions. Landfill disposal serves as a pragmatic and cost-effective waste management solution, making it particularly appealing to both developing and underdeveloped countries [34]. Its simplicity in implementation, requiring minimal specialized equipment and technology, facilitates swift waste removal, addressing immediate sanitation needs. Furthermore, landfills effectively reduce waste volume, a crucial benefit in densely populated areas with limited space. However, this approach comes with several significant disadvantages. Landfills can contribute to environmental pollution, releasing hazardous leachate and greenhouse gases, including methane, which fuels climate change [19]. Examining successful models from countries like China, India, and Brazil can provide insights into effective waste management and recycling strategies [24,27,29]. In developing countries, the recycling of food waste (FW) is regrettably underutilized. Several factors contribute to the inadequacy of recycling systems, including the absence of official policies to encourage public participation in recycling initiatives and the lack of effective incentives for FW recycling programs, as documented by Sarker, [1]. For instance, in many Latin American developing countries like Brazil and Mexico, the existing recycling systems are hindered by incomplete legislative frameworks. Additionally, these nations have lacking of comprehensive educational curriculums on the methodology of improving rates of separation as well as collection of FW. Moreover, limited involvement of private organizations in reutilizing of waste and diversion endeavors is also observed. In addition to this, insufficient funding is also noticeable fact for improving FW facilities [28]. To illustrate the contrast, Malaysia implemented household waste separation legislation in 2023, which included two separate bins for organic and recyclable waste, as reported by Lim et al., [27]. Thailand's government has also taken steps to promote FW separation at the source in select cities, aligning with their "3Rs" implementation aimed at increasing organic waste utilization by 50 % before 2026, as mentioned by Walter et al., [29].

Examining successful waste management and recycling models from countries like China, India, and Brazil can provide valuable insights into effective strategies [24,27,29]. However, in many developing countries, including several Latin American nations such as Brazil and Mexico, FW recycling remains underutilized due to various challenges. Sarker [1] documents factors contributing to inadequate recycling systems, such as the absence of official policies encouraging public participation and the lack of effective incentives for FW recycling programs. Additionally, incomplete legislative frameworks hinder existing recycling systems in these countries. Furthermore, there is a lack of comprehensive educational programs aimed at improving separation and collection rates of FW, along with limited involvement of private organizations in waste reuse and diversion efforts [28]. Insufficient funding also poses a significant challenge for improving FW recycling facilities. In contrast, recent initiatives in Malaysia and Thailand demonstrate progress in FW recycling. Malaysia implemented household waste separation legislation in 2023, requiring separate bins for organic and recyclable waste, as reported by Lim et al. [27]. Thailand's government is promoting FW separation at the source in select cities, aligning with their "3Rs" implementation aimed at increasing organic waste utilization by 50 % before 2026, as mentioned by Walter et al. [29]. These examples illustrate efforts to address challenges and promote effective FW recycling strategies, providing valuable lessons for other developing countries striving to improve waste management practices.

Open burning refers to the practice of igniting and combusting waste materials, including organic and non-organic materials, in an uncontrolled or informal setting, often in open air or makeshift burn pits. This method is typically used for waste disposal and includes the burning of various materials such as agricultural residues, construction debris, plastics, and even food waste. However, open burning is associated with several significant environmental and health concerns. The combustion of waste materials in open fires releases a wide range of pollutants into the atmosphere, including particulate matter, hazardous air pollutants (HAPs), volatile organic compounds (VOCs), and greenhouse gases [35]. These emissions can contribute to air pollution, adversely affecting air quality and human health. The fine particulate matter generated from open burning can penetrate deep into the respiratory system, leading to respiratory problems and other health issues. Furthermore, open burning is an inefficient method of waste disposal, as it often leaves behind unburned residues and can release toxins and harmful substances into the environment. Additionally, this practice can contribute to the generation of dioxins and furans, highly toxic and persistent organic pollutants with detrimental effects on both human and environmental health. Due to these environmental and health concerns, open burning is discouraged and regulated in many countries [34,35]. Governments and environmental agencies advocate for safer waste disposal practices, such as controlled incineration, landfilling, composting, and recycling, to minimize the negative impacts associated with open burning [36].

Composting is a sustainable and environmentally friendly waste management method that involves the natural decomposition of organic materials into nutrient-rich soil conditioner. This process harnesses the power of microorganisms, such as bacteria, fungi, and earthworms, to break down organic matter like food scraps, yard waste, and agricultural residues [34]. Composting typically takes place in controlled environments like compost bins or piles, where organic materials are layered and periodically turned to ensure adequate aeration and decomposition. During the composting process, microorganisms break down the organic matter, converting it into a humus-like substance known as compost. Compost offers numerous benefits. First and foremost, it provides an excellent soil conditioner, enriching soil with essential nutrients, improving its structure, and enhancing its water retention capacity. This, in turn, promotes healthier plant growth and increases agricultural productivity [37]. Additionally, composting diverts organic waste from landfills, reducing the generation of harmful greenhouse gases like methane. Composting is a sustainable practice that reduces waste, enriches soil, conserves resources, and supports a healthier environment. It is widely adopted in both residential and commercial settings, contributing to responsible waste management and sustainable agriculture practices. Composting is an effective method for food waste (FW) disposal in developing countries. In India, over 70 composting facilities process mixed municipal solid waste (MSW), recycling approximately 5.9 % of the total FW, yielding about 4.3 million tons of compost annually. Most of these facilities handle mixed waste, while a few, such as those in Vijayawada and Suryapet, manage source-separated organic waste. Thailand similarly employs composting for organic solid waste treatment, with around 0.59 million tons of FW being recycled as organic fertilizer and biogas. National strategies in some countries, like Thailand and Malaysia, emphasize composting to improve FW utilization. However, challenges persist due to unpurified waste feedstock resulting from incomplete source separation systems, leading to weak composting markets and competition with chemical fertilizers. Despite international NGO efforts to promote small-scale composting in developing countries, compost quality remains a concern, hindering recycling awareness and effectiveness [38].

In developing countries such as India, China, Brazil, Thailand, Malaysia, and Mexico, anaerobic digestion technologies play a crucial role in sustainable waste management and renewable energy production. These countries utilize anaerobic digestion to convert diverse organic waste sources, including agricultural residues and municipal waste, into biogas for energy generation. For example, India promotes decentralized biogas plants in rural areas, while China employs large-scale anaerobic digestion to treat organic waste efficiently. Brazil focuses on biogas production from agricultural and urban waste, supporting energy needs in rural communities. Thailand and Malaysia leverage anaerobic digestion for biogas generation from industrial waste like palm oil effluent, reducing reliance on fossil fuels. Mexico adopts anaerobic digestion to manage organic waste comprehensively, contributing to waste-to-energy initiatives and methane emission reduction. These initiatives underscore the increasing importance of anaerobic digestion in developing countries, fostering sustainable waste management and renewable energy transitions. Anaerobic digestion represents an advanced approach to waste management and the generation of renewable energy. It harnesses the natural decomposition process driven by microorganisms in an oxygen-deprived environment. This biological process effectively breaks down organic materials like food waste, agricultural residues, and wastewater sludge into biogas, primarily composed of methane and carbon dioxide, along with a nutrient-rich substance known as digestate. Anaerobic digestion occurs within sealed, oxygen-free reactors or digesters where anaerobic microorganisms thrive. In this process, organic waste is mixed with water and introduced into the digester, creating an environment devoid of oxygen. Anaerobes work to break down complex organic compounds into simpler substances through a series of biochemical reactions. This microbial activity results in the production of biogas, a valuable renewable energy source that can be employed for electricity generation, heating, or as a vehicle fuel. The remaining digestate can serve as a nutrient-rich fertilizer for agricultural applications. Anaerobic digestion offers numerous advantages, including waste reduction, the generation of renewable energy, and the reduction of greenhouse gas emissions. It provides an environmentally friendly solution for handling organic waste, diverting materials from landfills, and lessening the environmental impact of waste disposal. This technology plays a pivotal role in the shift towards sustainable waste management and renewable energy production while contributing to a circular economy by recycling valuable nutrients from organic waste streams [39]. Since 2023, anaerobic digestion (AD) has gained widespread adoption for food waste utilization in European countries and several developed nations in Asia. However, in many developing nations, AD remains under-utilized for FW managing. In China and India, many institutes and NGOs have initiated small-to large-scale anaerobic digesters to develop AD technology for FW treatment. While India has piloted AD and opened biogas plants, China is preparing or implementing about twenty projects involving co-fermentation AD with municipal solid waste, FW, and manure. Nonetheless, many of these digesters may face functionality challenges because of methodological issues, inadequate operations, or management regulations. In, Philippines, Vietnam, Indonesia, AD is integrated with composting for FW disposal in landfill sites, while Jamaica and Thailand have made significant strides by combining AD and aerobic composting processes for FW treatment [40].

Informal recycling and scavenging represent grassroots and often unregulated methods of resource recovery and waste management. These practices involve individuals or small groups collecting, sorting, and selling recyclable materials, often from municipal waste streams, without formal employment or oversight. Informal recycling is a vital component of many developing countries' waste management systems, contributing to resource conservation, poverty alleviation, and waste diversion from landfills. In informal recycling, individuals typically focus on specific materials, such as paper, cardboard, plastics, metals, or glass, which they gather from waste bins, landfills, or streets. These materials are then sorted, cleaned, and prepared for sale to recycling facilities. Scavengers, on the other hand, may collect a broader range of items, including reusable goods or items with intrinsic value, such as electronics or appliances [41]. While informal recycling and scavenging play essential roles in waste reduction and recycling, they often operate in challenging and hazardous conditions. Workers are exposed to health and safety risks, including exposure to hazardous waste and inadequate protective gear. Additionally, these practices can contribute to social and environmental challenges, such as child labor, exploitation, and unregulated waste handling. Efforts are underway to formalize and improve informal recycling practices by providing training, safety equipment, and legal recognition for these workers. Recognizing the value of informal recycling within waste management systems is crucial for achieving more sustainable and inclusive waste management practices while addressing the unique challenges faced by those involved in these activities [42].

The generation of biogas is a sustainable and renewable energy production method that leverages the natural decay of organic materials within an oxygen-deprived setting to yield biogas. This biogas primarily comprises methane (CH4) and carbon dioxide (CO2) and serves as a clean and versatile energy source. Typically, biogas production takes place within anaerobic digesters or biogas facilities, where microorganisms decompose organic matter, including food waste, agricultural residues, and sewage sludge, through a sequence of biochemical reactions [41,42]. In the course of anaerobic digestion, microorganisms break down the organic materials into simpler compounds. This microbial process generates biogas as a byproduct, which can be captured and stored for diverse applications. Biogas can be employed for electricity generation, heating, and as a substitute for natural gas. Furthermore, the remaining residue from the digestion process, known as digestate, contains valuable nutrients and can be utilized as an effective fertilizer for agricultural purposes [42]. Biogas generation yields numerous environmental and economic advantages. It reduces the environmental impact of organic waste disposal by transforming it into a valuable energy source, thus mitigating greenhouse gas emissions and decreasing dependence on fossil fuels. Biogas also offers a dependable and sustainable energy supply, particularly in rural areas with limited access to electricity. In essence, biogas generation embodies an environmentally friendly and circular approach to managing organic waste while concurrently generating renewable energy, contributing to a more sustainable and resource-efficient future [43].

The utilization of FW in the form of animal and fish feed is a sustainable and resource-efficient practice that helps mitigate waste generation while providing valuable nutrition to animals in various sectors of agriculture and aquaculture. This approach involves collecting and processing FW to create feed products suitable for livestock, poultry, and fish. FW can encompass a range of materials, including food scraps, agricultural surplus, and by-products from food processing industries [[36], [37], [38], [39], [40]]. To create animal and fish feed from FW, it typically undergoes various treatments such as drying, grinding, and mixing to enhance its nutritional value and safety. These processed feeds can replace or supplement conventional feed ingredients like grains and soybeans, reducing the environmental footprint of animal agriculture by diverting FW from landfills or incineration. While this practice offers several advantages, including waste reduction, cost savings for farmers, and reduced reliance on traditional feed sources; it also comes with challenges related to the quality and safety of FW-based feeds. Ensuring that the feed meets nutritional requirements, adheres to safety standards, and does not pose health risks to animals or consumers is essential [41]. Production of animal and fish feed from FW presents an opportunity to create a circular and sustainable food system by valorizing food waste and reducing the environmental impact of livestock and aquaculture industries. To realize these benefits, effective regulations, quality control measures, and education about safe feed practices are crucial [44].

Community-based initiatives are grassroots efforts driven by local residents, organizations, or groups to address specific social, environmental, or economic challenges within their communities. These initiatives typically involve collective action, cooperation, and a shared sense of responsibility to bring about positive change. They can take a wide range of forms, from environmental conservation projects and waste reduction campaigns to community gardens, renewable energy cooperatives, and social programs aimed at improving the well-being of community members [45]. One of the key features of community-based initiatives is their strong focus on local needs, priorities, and resources. These efforts often arise in response to unique challenges or opportunities within a community and are tailored to suit the local context. Community members actively participate in planning, implementing, and maintaining these initiatives, fostering a sense of ownership and empowerment. Community-based initiatives offer several benefits, including building social cohesion, promoting sustainability, and enhancing the resilience of communities. They empower individuals and groups to take control of their circumstances, leading to positive changes in various aspects of community life [43]. Additionally, these initiatives can serve as models for broader social and environmental change, inspiring similar efforts in other regions. While community-based initiatives can be highly effective, they may face challenges related to funding, capacity building, and sustainability. Support from local governments, NGOs, or external stakeholders can play a crucial role in helping these initiatives thrive and achieve their goals. Overall, community-based initiatives exemplify the power of local action and collaboration in addressing complex issues and fostering positive change within communities, making them an essential component of sustainable development and community empowerment [46].

### 2.4 Food waste policies: developed vs developing countries

Food waste management policies and regulations vary widely between developed and developing countries due to differences in infrastructure, economic capacity, and resource availability. In developed nations, comprehensive regulations are in place to address food waste, including guidelines for labeling, donation, and disposal. Some countries even have laws requiring supermarkets to donate unsold food. Additionally, developed countries set specific targets for food waste reduction and conduct public awareness campaigns to educate the public about responsible consumption. In contrast, many developing countries face challenges in implementing formal food waste policies due to limited infrastructure and resources [47]. However, some are making efforts, often with international assistance, to improve food waste management through local initiatives and capacity-building programs. The situation is dynamic, highlighting the need for continued international collaboration to bridge the gap in food waste management practices. In the context of food waste management policies, the disparities between developed and developing countries run deeper than just regulatory differences. Developed countries benefit from established waste management infrastructure, including efficient collection systems and advanced recycling and disposal facilities. This enables them to implement and enforce comprehensive regulations effectively [48]. In contrast, many developing nations struggle with inadequate waste management infrastructure, limited financial resources, and competing priorities, such as addressing food insecurity and poverty. In developing countries, informal recycling and waste-picking practices often emerge as a pragmatic response to the lack of formal waste systems. While these activities contribute to some degree of food waste reduction and recycling, they are accompanied by safety and health risks for informal workers. Efforts are being made in some developing countries to address food waste. International organizations and NGOs play a crucial role in providing technical assistance and capacity-building support to help these nations improve their food waste management practices [49]. Some recent policies and regulations related to FW management in select countries are outlined in Table 3 and Table 4.

**Table 3:** Food waste policies: Developed countries.

Country/region	guidelines	Year of Publication	Main Contents
EU	(i)Circular economy package	2015	Offer a variety of procedures to lessen waste of food.
	with strategies on food donation.	2017	Focusing standardize food preservation process.
	(ii) Waste framework directive	2018	Revisions turn around enriching food preservation.
	developed on food losses and waste	2019	Establishment of the "EU platform on food loss and food waste."
	green deal of Europe	2020	Strategic objectives include "revision of EU food date marking rules, legal constraints on food waste."
	Redistribution of food in Europe	2020	An exhaustive examination on food endowment guidelines covering hygiene, classification, accountability, levy.
UN	Agreement on food security	1974	The conception of "food security" was announced with regulations of relevant features.
	Convention on biological diversity	1992	These regulations emphasize food's crucial role in human survival, health, and the urgency of combatting food waste for sustainable development.
	Rome declaration with action plan	1996	It advocates the right to food and urges governments to legislate responsibilities for food security encompassing poverty reduction, industrial growth, production enhancement, and waste reduction.
	Think.Eat.Save	2013	Highlighting environmental complications triggered by FW and requesting for food preservation.
	The 2030 agenda for sustainable development	2015	SD goal is set targeting reduction of food loss (worldwide) to half by 2030
	The Paris agreement	2016	Propositions to prioritize hunger eradication, food security
Germany	National strategy to reduce food waste	2019	Establish goals to cut retail and consumer food waste in half by 2030, reduce waste in production and supply chains, and designate food waste reduction as a national priority.
Norway	Agreement to reduce food waste	2017	It mandates food stakeholders, including producers, retailers, households, and government agencies, to collectively shoulder the responsibility for curbing food waste, fostering conservation efforts, and combatting its prevalence.
UK	National waste lessening policy	2007	Highlighting economic as well as ecological effect of decreasing FW.
USA	Agreement between FDA, EPA, USDA for cooperation and management to lessen FW	2018	Improving cooperation among federal organizations for educating Americans to reduce loss and waste of food and, stimulating food preservation practice.
Argentina	National program on loss, waste reduction of food	2019	Refining guidelines of food aids, execution procedure, etc.
Korea	FW reduction master plan.	1996	Guidelines for FW reprocessing
Japan	The act for promoting reduction of loss of food and waste	2019	Clarifying Government's accountability for avoiding FW

**Table 4:** Food waste policies: Developing countries.

Countries (regions, institutions)	Laws and regulations and related documents	Year of Publication	Main Contents
Brazil	Providing for combating food waste and donating surplus food for human consumption	2020	This law establishes precise regulations for reducing food waste and facilitating safe surplus food donations, ensuring that donated items, even with damaged packaging, maintain quality and safety standards, and must be provided at no cost to individuals or groups experiencing food insecurity or nutritional risks.
Turkey	Environmental Law	2011	These regulations constitute a comprehensive framework encompassing various waste categories, including packaging, batteries, oils, hazardous substances, and more, to promote sustainable waste management practices and environmental protection.
Malaysia	Solid Waste and Public Cleansing Management Act		This Act is designed to establish and oversee the management of controlled solid waste and public cleansing, aiming to uphold sanitation standards and address related matters.
China	Anti-food Waste Law of China.	2021	This law provides clear definitions for 'food' and 'food waste,' assigns responsibilities to various stakeholders, promotes responsible consumption, and establishes a reporting mechanism for food waste violations.
Thailand	General Food Safety Regulations in Thailand	2022	The Food Act B.E. 2522 (1979) serves as Thailand's primary legislation for food safety, encompassing quality control, licensing, consumer safety, and oversight by regulatory bodies, with additional regulations on prohibited foods, standards, labeling, additives, and related aspects.
India	Waste Management Laws in India		India's waste management is crucial for environmental sustainability, governed by various regulations, including the Environmental Protection Act, Hazardous Wastes Rules, Plastic Waste Rules, Bio-Medical Waste Rules, E-Waste Rules, and Batteries Rules, ensuring proper waste handling and protection of the environment.
Bangladesh	National 3R Strategy for Waste Management	2010	In urban Bangladesh, waste management consists of formal city authorities, community-driven initiatives, and informal workers, with food waste often ending up in landfills, open burning, or dumping, highlighting the need for innovative, sustainable management solutions and research to harness its bioresource potential.
South Africa	National Waste Management Strategy	2020	The strategy underscores waste reduction, recycling, collaboration with diverse stakeholders, and alignment with sustainable development goals to enhance environmental protection and socio-economic well-being.
Indonesia	The Act Regarding Waste Management	2008	Managing waste is steered including accountability, sustainability, viability, fairness, consciousness, togetherness, protection, safekeeping, and financial assessment.

Pilot programs, community initiatives, and awareness campaigns are emerging, demonstrating that there is a growing recognition of the importance of food waste reduction in these regions. However, the path toward effective food waste management in developing countries is multifaceted and challenging. It requires not only the establishment of policies and regulations but also investments in infrastructure, education, and sustainable practices. Bridging the gap between developed and developing countries in this regard requires a coordinated effort from governments, international organizations, and local communities to create tailored solutions that align with the unique socio-economic and environmental contexts of each nation [[47], [48], [49]]. In many developing countries, the practice of FW recycling is not yet widespread, and regulations pertaining to FW management remain incomplete. Consequently, a significant portion of FW ends up mixed with municipal solid waste (MSW) and is ultimately landfilled. For instance, Thailand has established relevant regulations through the National 3Rs Strategy (2011) and the National Climate Change Strategy (20011-2023). These policies set ambitious national targets for FW reduction, aiming for reductions of 5 %, 30 %, and 50 % by 2016, 2021, and 2026, respectively. Brazil drafts waste law targeting significant reductions in landfills waste including dry recyclable as well as damp organic waste, aiming for reductions of about 36 % and 53 % by 2031 [50]. Turkey has implemented law for reduction landfilling by FW increasing composting capabilities to produce methane gas for generating electricity in line with the directive of EU landfill by 2025. Malaysia has made positive strides in FW management through implementing SWPCM Act, 2007 emphasizing the 3Rs. China also has adopted strategies and regulations for FW management, but coordination among government ministries and agencies remains a challenge [50]. In the given passage, a number of nations, including Bangladesh, Pakistan, Thailand, Indonesia, India, and Ukraine, have collaborated with non-governmental organizations (NGOs) to initiate initiatives and campaigns with the goal of advocating for eco-friendly solutions for food waste (FW) such as composting and anaerobic digestion (AD), or enhancing public knowledge about FW management [41].

Nevertheless, these areas typically lack comprehensive or fully developed regulations at the national level for managing food waste. While some developing countries have shown interest in addressing FW management, comprehensive legal stipulations remain incomplete or unapproved. Many of these nations rely on overseas aid budgets, often from NGOs and the World Bank, to fund recycling activities and projects, as they allocate limited budgets for segregating activities and establishing FW treatment facilities [48].

The experiences of developed countries underscore that a government must establish specific FW reduction objectives and enact comprehensive legislative regulations to effectively address FW management challenges. The main obstacles in many developing countries include inadequate administrative measures and insufficient budget allocation for enhancing recycling activities in the FW management process [51].

## 3 Literature review methodology

**Fig. 4:**
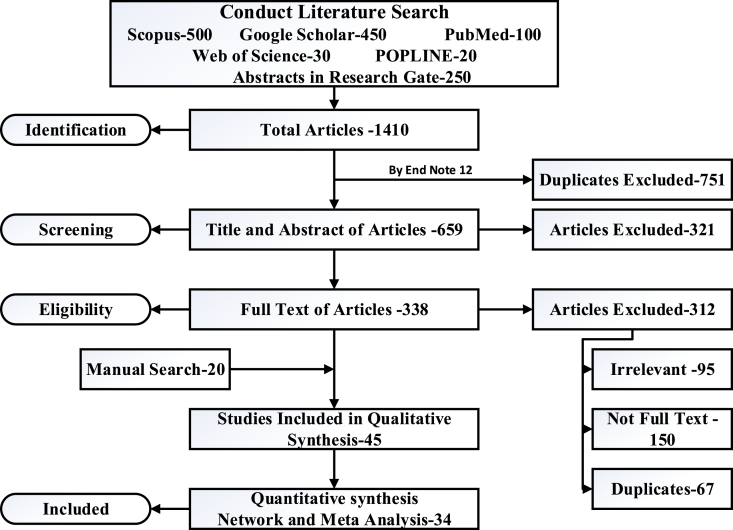
Preferred Reporting Items for Systematic Reviews and Meta-Analyses flow diagram of studies' screening and selection.

Systematic reviews and meta-analyses are essential methods in evidence-based research that synthesize existing literature effectively. They begin with a focused research question and employ a comprehensive search strategy across databases to identify relevant studies meeting predefined criteria. Each study undergoes critical appraisal to assess quality and relevance. Originally designed for navigating food waste management in developing countries, the Preferred Reporting Items for Systematic Reviews and Meta-Analyses (PRISMA) guidelines have gained broad acceptance across various fields conducting evidence synthesis studies. These guidelines offer researchers a structured framework to ensure that their systematic review or meta-analysis is conducted and reported in a rigorous and comprehensive manner. The preliminary search conducted on January 01, 2022, yielded a total of 1410 articles. This study employed the PRISMA procedure, as outlined in Fig. 4, which also illustrates the number of articles retrieved from each respective database. Navigating the contemporary landscape of food waste management in developing countries presents a complex and multifaceted challenge. To comprehensively understand this issue, a systematic literature review is essential. This review aims to provide a detailed overview and prospective analysis of food waste management practices, challenges, and opportunities within developing countries over the past decade.

By defining clear research objectives and scope, the review focuses on identifying relevant studies from academic databases, government reports, and NGO publications. Key search terms include variations of "food waste management in developing countries" to capture a diverse range of perspectives. The literature screening process involves meticulous selection based on predefined inclusion criteria, ensuring that only studies directly related to food waste management in developing countries are included. Through data extraction and synthesis, common themes, trends, and insights will be identified, shedding light on prevalent challenges and innovative strategies adopted by developing nations. The analysis critically evaluates the quality and reliability of reviewed literature while acknowledging limitations and gaps for further research. Ultimately, the prospective analysis will provide actionable recommendations for policymakers, practitioners, and researchers to enhance food waste management practices and promote sustainability in developing countries. The findings of this review are documented in a comprehensive report, contributing valuable insights to the global discourse on sustainable food systems and waste reduction initiatives. Two independent reviewers meticulously examined the titles and abstracts of all papers, resulting in the selection of 38 articles for a comprehensive full-text review. After applying the criteria for full-text review, a total of 45 articles met the inclusion criteria, and an additional 20 articles were identified through a supplementary search strategy within this final stage. As a result, 34 articles were found to meet the criteria and were selected for inclusion. This additional search involved a thorough examination of the references cited within the 45 selected articles.

## 4 FW management in developing countries: benefits, drawbacks, and solutions

In the context of developing countries, the management of FW is a multifaceted challenge with both advantages and disadvantages. On the positive side, efficient FW management can yield significant environmental benefits by reducing greenhouse gas emissions associated with landfilling [34]. Additionally, it conserves valuable resources, offers economic opportunities through recycling and composting, and contributes to food security by redistributing surplus food to vulnerable populations. However, the lack of adequate infrastructure, limited financial resources, and informal waste picking practices pose significant disadvantages. To address these challenges, developing countries can adopt policies and regulations that guide FW reduction and safe disposal, invest in essential waste infrastructure, raise public awareness about responsible consumption, and seek international assistance from organizations and NGOs. By implementing these remedies, developing nations can move toward more sustainable FW management practices and unlock the benefits it offers while mitigating its drawbacks [52].

### 4.1 FW management in developing countries benefits

Firstly, in Bangladesh and South Africa, a notable practice involves food auditing conducted by various global food donors. These donors encompass a wide range of stakeholders, including agricultural producers, food manufacturers, retailers, government agencies, and individuals. This collaborative effort is carried out within the framework of the Global Food-bank Network (GFN), an organization dedicated to poverty relief and charitable initiatives. One of the primary objectives of this food donations platform is to prevent food wastage by redistributing surplus or leftover food to those in need. This approach has demonstrated significant potential, with the capacity to save an estimated 15.6 and 10 million tons of food annually [53]. The success of this platform in Bangladesh and South Africa highlights its potential as a model to be expanded to other countries, regardless of their development status. By extending this practice globally, both developed and developing nations can contribute to reducing food waste while simultaneously addressing issues of hunger and food insecurity. The collaborative and cross-sectoral nature of this approach underscores its effectiveness in optimizing food resource utilization and promoting sustainability in the global food supply chain. Furthermore, the experiences of Thailand, Turkey, and Malaysia underscore the pivotal role that the private sector can assume in the realm of food waste (FW) recycling. Notably, these countries lack comprehensive government rules or policies governing FW management. Instead, the private sector has stepped in, making substantial contributions to addressing this issue. For instance, the CaribShare Biogas initiative in Jamaica represents a pioneering solution in this context. This private-sector-driven endeavor not only enables Jamaica to manage and dispose of organic waste in an environmentally sustainable manner but also offers a valuable source of clean energy [54]. This case highlights the potential for private sector innovation to fill gaps in FW management where government regulations may be lacking. It demonstrates the capacity of entrepreneurial initiatives to not only reduce food wastage but also generate positive outcomes such as renewable energy production. This example encourages other countries, both developed and developing, to explore partnerships and collaborations with private enterprises to enhance their FW recycling efforts and drive sustainable solutions that benefit both the environment and society [55].

Additionally, it's worth noting that in many developing countries, there is a growing recognition within current municipal solid waste (MSW) management policies of the importance of addressing food waste (FW) issues. This represents a significant step forward as it indicates a shift towards acknowledging and considering FW solutions within the broader waste management framework. As a promising development, governments are increasingly inclined to introduce specific management policies dedicated to FW or integrate FW management regulations as a distinct section within the overarching MSW management system. This strategic approach offers several advantages. By delineating FW management within the existing waste management policies, governments can provide a clear and structured framework for addressing FW-related challenges. This can encompass guidelines on FW reduction, diversion, recycling, and safe disposal practices. Moreover, such integrated policies can help streamline efforts, allocate resources effectively, and promote a more comprehensive and coordinated approach to FW management [56]. This is particularly beneficial as it recognizes the unique characteristics and requirements of FW within the larger waste management landscape. Overall, it signifies a positive shift towards more efficient and sustainable FW management practices in developing countries. Furthermore, another valuable approach to food waste (FW) management, especially in regions with temperate climates conducive to extended operation, involves small-scale anaerobic digestion (AD) facilities. This method proves particularly suitable for large, geographically dispersed areas, as exemplified by India. India's vast landscape, with significant distances between communities, makes AD technology a favorable solution for decentralized FW treatment [56]. In specific regions of India, such as the southern and western parts, approximately 20,000 households have adopted biogas units equipped with AD technology to address FW challenges while simultaneously generating biogas [57]. This approach yields multiple advantages. First, it reduces the costs associated with collecting and transporting FW, making it a cost-effective solution. Second, it plays a crucial role in mitigating greenhouse gas (GHG) emissions, aligning with environmental sustainability goals. The success and applicability of small-scale AD facilities in India demonstrate their potential for broader adoption in various countries facing similar challenges. By offering both economic and environmental benefits, this approach could be scaled up and promoted on a more extensive basis, offering an effective solution to FW management that aligns with sustainability and climate objectives. This decentralized model has the potential to make a significant impact on reducing FW and its associated environmental footprint in developing nations [57].

In the near future, the evolution of innovative technologies promises to play a pivotal role in tackling the food waste (FW) challenge. Furthermore, the exchange of technologies and the assimilation of lessons learned from developed countries offer developing nation's valuable shortcuts in comprehending and enhancing their FW management systems. This knowledge transfer can significantly expedite progress. For instance, consider the case of China, which has embraced biochemical processes as cutting-edge technology to address FW issues in major cities such as Beijing, Shanghai, and Guilin [28]. This adoption of advanced methods signifies the potential for technology-driven solutions to FW problems in developing nations. Similarly, Thailand has set ambitious targets for FW utilization, aiming to achieve a 50 % utilization rate by 2026. This goal is pursued through the implementation of an integrated biodigester system for sorting organic waste and the application of mechanical-biological treatment for unsorted organic waste [55,56]. Such endeavors highlight the significant role that technological advancements can play in driving FW management improvements in developing countries. As technology continues to evolve and mature, it offers promising avenues for addressing FW challenges effectively and sustainably. Developing nations can leverage these innovations not only to reduce FW but also to harness its potential benefits, contributing to a more efficient and environmentally friendly FW management landscape [58].

### 4.2 FW management in developing countries drawbacks

Food waste (FW) management in developing countries presents a range of challenges. One significant hurdle is the lack of adequate infrastructure, including waste collection and disposal systems, which can lead to improper FW disposal in open dumps or landfills [57]. Additionally, limited financial and human resources constrain investments in advanced technologies and comprehensive waste reduction programs. Informal waste picking is prevalent in some regions, contributing to inefficient FW handling. Inadequate regulations specific to FW management and low public awareness about responsible consumption further compound the problem [55]. Concerns about food security and limited access to advanced FW management technologies also hinder progress. Health and environmental risks associated with improper disposal methods pose additional challenges, as do cultural and behavioral factors influencing FW generation. Economic pressures often overshadow efforts to prioritize FW reduction. Overcoming these disadvantages requires holistic strategies encompassing regulatory frameworks, infrastructure development, awareness campaigns, and technology adoption, with international cooperation playing a vital role in supporting these efforts [59].

Budget allocation for the management of municipal solid waste (MSW) varies significantly between less developed and more developed nations, as demonstrated by World Bank estimates. In less developed countries, a significant proportion (around 80 %) of their budget is channeled into waste collection and landfill operations. Conversely, in developed countries, less than 10 % of their overall budget is dedicated to collection, with a larger share of funds directed towards sorting activities, establishing treatment facilities, and implementing waste management initiatives designed to increase public awareness of waste recycling and recovery [[57], [58], [59]]. As a result, less developed countries often grapple with regulatory mismanagement, leading to the disposal of unsorted food waste (FW) in landfills without proper separation. Furthermore, many of these nations have not received sufficient attention from key stakeholders responsible for addressing FW-related concerns [60]. Consumer behavior also significantly impacts FW generation, particularly during the consumption phase. Research suggests that in developed countries with a high level of education, FW tends to be generated “before the meal” as people are more conscientious about meal planning. In contrast, in settings with lower and middle education levels, which are common in developing countries, FW tends to occur 'after the meal. This difference is attributed to people in developing countries generally paying less attention to how their FW will be disposed of, leading to wasteful consumption habits [59]. In developed countries, there is a greater awareness of the importance of minimizing food waste, which influences consumer behavior. However, in many developing countries, unsold commodities and FW from restaurants, markets, or retail outlets are often directly treated as part of municipal solid waste, as there are limited guidelines or regulations to encourage saving and recycling of FW [61].

Furthermore, the mere promulgation of FW management policies without effective enforcement by governments can result in limited practical implementation. This challenge is evident in countries like China and India, where policies have been established but may not be rigorously enforced, leading to gaps in wide-scale application and effectiveness [28]. It underscores the importance of not only creating regulatory frameworks but also ensuring their consistent enforcement to drive meaningful change in FW management practices [62].

### 4.3 FW management in developing countries solutions

**Fig. 5:**
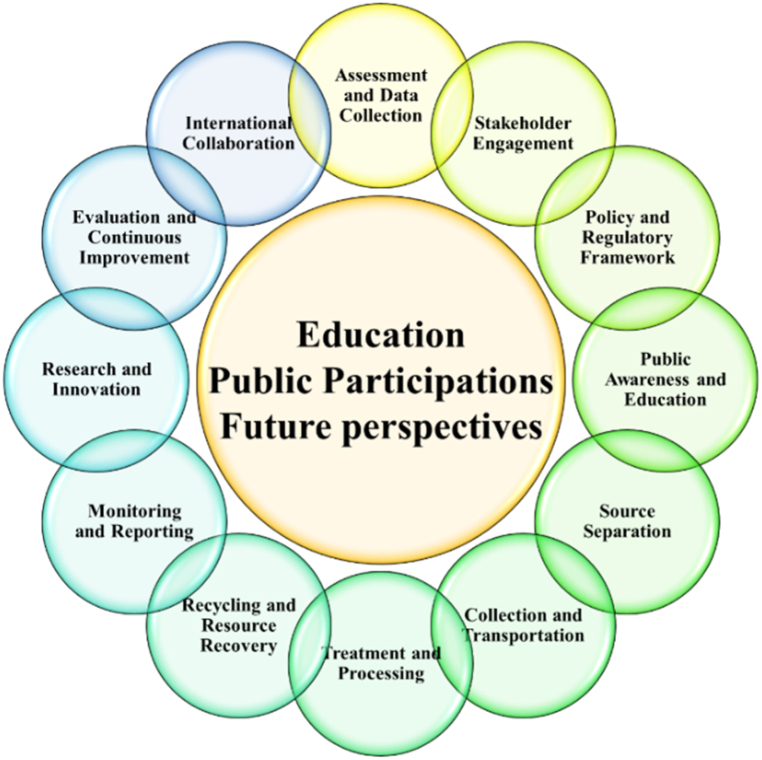
Key steps of designing and integrated FW management system.

Addressing the complex issue of FW management in developing countries necessitates a comprehensive and adaptable approach [49]. Key strategies include the development of infrastructure, the establishment of effective regulatory frameworks, and fostering awareness through education campaigns. Private sector engagement and community-based initiatives can play pivotal roles, as can the transfer of relevant technologies from developed countries. International cooperation, behavioral change initiatives, and research-driven policymaking are all essential components of an effective remedy [63]. By combining these strategies and tailoring them to local conditions, developing countries can make significant strides in mitigating food waste and its associated environmental, economic, and social challenges. Moreover, it's crucial to recognize that food waste management is not solely a matter of environmental concern but also a social and economic one. Effective remedies should address the link between food waste and food insecurity [44]. Surplus food that is safe for consumption but not sold can be redistributed to vulnerable populations through food banks and charities. This not only reduces waste but also contributes to alleviating hunger and poverty. Additionally, building partnerships between governments, non-governmental organizations (NGOs), and the private sector is instrumental in implementing these remedies. Collaborative efforts can mobilize resources, expertise, and knowledge sharing to create sustainable and scalable solutions. Furthermore, monitoring and evaluation mechanisms are essential to track progress and adjust strategies as needed [51]. Regular assessments of food waste generation, reduction, and management should inform policy adjustments and the allocation of resources. In conclusion, tackling food waste in developing countries requires a holistic approach that considers environmental, social, and economic dimensions. By implementing these multifaceted remedies and fostering collaboration among various stakeholders, developing nations can reduce food waste, promote sustainability, and address the broader challenges of food security and poverty alleviation [58]. To illustrate the holistic approach to designing an integrated FW management system, refer to Fig. 5. This framework ensures that FW management addresses public education, future considerations, and community involvement at every stage of the process. Drawing from our prior analysis of the advantages and disadvantages of FW treatment, in this paper, we present a series of remedies aimed at mitigating drawbacks and facilitating the implementation of FW management strategies in developing nations [53]. These seven proposed solutions are vital for enhancing FW management systems in these regions. In some developing countries, like Malaysia and Thailand, existing FW management systems suffer from budget constraints [[52], [53], [54]]. To address this, governments should prioritize the integration of FW management with municipal solid waste (MSW) management. This entails establishing formal collection sectors with dedicated budgets and infrastructure support. Many developing countries allocate a significant portion of their budgets to landfilling FW, leaving limited resources for recycling efforts. Governments must incentivize the adoption of innovative technologies like anaerobic digestion and biochemical treatment to enhance FW recycling efficiency.

Legislation and business practices should align with sustainable food production principles, such as the closed-loop supply chain model [44]. This shift is essential to reduce FW generation and encourage responsible practices throughout the food supply chain. Governments should establish national food banks and collaborate with international organizations like the World Bank to create a global food donation platform. This initiative can divert surplus food, both post-harvest losses from farmers and unsold food from retailers and wholesalers, to address hunger and manage FW effectively [64].

Current policies in countries like China and Vietnam address waste broadly, lacking specific adaptations for FW. To improve the situation, governments should develop tailored FW policies, encourage collaboration among relevant ministries, and consider legislation to prohibit FW disposal in landfills [28]. Encouraging the growth of FW recycling markets, especially in agriculture-dependent economies like Indonesia, Thailand, and Vietnam, is crucial. Governments can provide financial incentives to support domestic FW recycling facilities and promote bio-composts and bio-fertilizers. In livestock-dependent nations like China, India, and Mexico, low-cost FW treatment through animal feeding should be promoted. This approach not only addresses feed costs but also aids in mitigating climate change through FW disposal [49]. Enhancing community awareness and shifting consumer behavior to reduce FW production is imperative. Governments should support campaigns and projects like "Food Waste into Energy" (in the United States), "Love Food-Hate Waste" (in the United Kingdom), and the "Zero Waste" strategy (in Europe) to raise awareness and encourage responsible practices. Governments should consider key factors when designing FW management systems, including reviewing existing systems, regulations, and technologies. At the national level, they should set standards, allocate funding, and establish short- and long-term FW management targets. At the local level, roles and responsibilities of stakeholders need clarity. The iterative nature of FW management requires continuous adjustment and community engagement throughout the process [65].

## 5 Key insights from managing FW in developing countries

Establishing an integrative FW management system in developing countries is a multifaceted endeavor that demands a coordinated effort from various stakeholders. The initial steps involve assessing the current food waste landscape and crafting robust policies and regulations aligned with international standards. Engaging government agencies, local authorities, businesses, and the public is paramount, with public awareness campaigns playing a pivotal role in fostering a culture of waste reduction [58]. The system must include mechanisms for food redistribution, setting reduction targets, and separating food waste at its source. Investing in composting, anaerobic digestion, and waste-to-energy facilities can effectively process organic waste. Monitoring, reporting, and research are essential for continuous improvement, while capacity building ensures that personnel are equipped to handle food waste efficiently. Incentives and penalties encourage participation and compliance, while international collaboration can offer valuable insights. Regular review, transparency, and public reporting will help developing countries make significant strides in reducing food waste and promoting sustainability [66].

### 5.1 Current Situation of FW Management in Taiwan

**Fig. 6:**
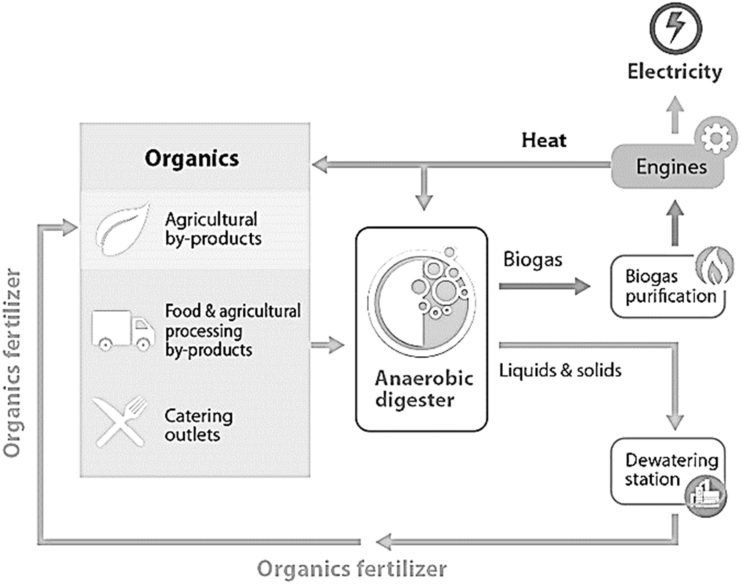
Current situation of FW management in Taiwan.

The case study from Taiwan provides valuable lessons on the successful establishment of an integrated FW controlling method, particularly in the context of a high-income country. Taiwan's achievement in effective waste managing can be accredited to a combination of integrated technologies and robust government enforcement of appropriate policies. This model of FW management in Taiwan holds promise for addressing similar challenges in developing countries [67]. For example, Costa Rica has looked to the Taiwanese experience as a reference point for improving its own waste management system [68]. The setting up development of Taiwan's integrated FW controlling system is summarized in Fig. 6. Taiwan's success can be attributed to several key factors such as a command-and-control techniques incentives, and mixture of education.

Taiwan has employed a multifaceted approach that includes educating the public, providing incentives for responsible behavior, and enforcing regulations when necessary. This holistic strategy has contributed to their success in managing FW effectively. Taiwan has devised an economic mechanism that incentivizes environmentally friendly practices. This approach not only profits the environment but also provides benefits to municipalities [69]. It aligns economic interests with environmental goals, motivating stakeholders to actively participate in FW management efforts. Since 2001, the Taiwanese EPA has implemented kitchen waste assemblage and reutilizing plans nationwide. These plans have been extended from homegrown cities to major municipalities, demonstrating a commitment to expanding and enhancing kitchen waste reutilizing determinations [70]. Taiwan's success in establishing an integrative FW managing system underscores the importance of a multifaceted approach that includes education, incentives, and regulatory enforcement. Moreover, their flexible economic mechanism and commitment to expanding and improving waste management programs have yielded positive results. Fig. 6 depicted that the current situation of FW management in Taiwan. Taiwanese model provides valuable insights for other countries, particularly those in the process of addressing FW challenges, whether they are high-income nations like Taiwan or increasing nations looking to progress their waste controlling methods [69]. In 2002, Taiwan initiated a groundbreaking program known as the 'Total Reutilizing for Kitchen Garbage' package, which aimed to systematically isolate and collect FW from various sources, including restaurants, hotels and residential areas [71]. This collected FW was then put to good use, with 80 % of it being repurposed as animal feed and the remaining 20 % transformed into valuable fertilizer [71]. This marked a significant step toward more sustainable waste management practices in the country. Over time, Taiwan saw a gradual annual increase of approximately 5 % in its total waste recycling rate, with the ambitious goal of reaching a 35 % recycling rate. In 2003, the national FW recycling program officially became a part of the Executive Yuan, and the Environmental Protection Administration (EPA) allocated budgetary resources to support nationwide FW recycling efforts [70]. This commitment from the government was instrumental in advancing FW recycling initiatives. From 2003 to 2008, Taiwan's EPA implemented a comprehensive program called the 'National Development Plan - Green Industry' with the aim of creating incentive mechanisms to stimulate both the industrial and private sectors to establish FW recycling and reutilization facilities, as documented by Thi et al. [70]. These incentives played a pivotal role in fostering widespread participation in FW recycling initiatives. Currently, the national FW recycling program has been integrated into a more extensive effort referred to as the 'Public Development Program - General Waste Resource Recycling Promotion Program. This consolidation is part of Taiwan's broader strategy to ensure environmentally responsible handling of FW and aligns with the overarching 'Zero Waste' policy [[70], [71], [72]]. In essence, Taiwan's journey in developing and expanding its FW recycling programs highlights the significance of strategic planning, government commitment, and incentive structures in achieving sustainable waste management practices. The country's success in recycling FW not only reduces environmental impacts but also demonstrates a commitment to responsible resource utilization, setting an example for others seeking to address FW challenges [72].

Moreover, Taiwan has implemented an effective recycling fund known as the ‘Taiwan EPA's Recycling Funds’. This fund serves as financial support for licensed collectors and recyclers, facilitating the enhancement of food waste (FW) segregation, collection, and recycling activities. This financial incentive system encourages and empowers key stakeholders in the waste management process to actively participate in FW recycling initiatives [73]. To bolster the resources available in the recycling fund, the Taiwanese government introduced the 'Waste Disposal Act.' Under this legislation, residents are mandated to recycle twelve specific types of waste items. This comprehensive approach not only encourages individual and manufacturer compliance but also imposes penalties on those who fail to adhere to the recycling regulations. Consequently, residents and manufacturers who do not comply with the rules face fines, and waste-collection crews have the authority to refuse the collection of their waste. This command-and-control strategy in FW management has proven to be highly effective in ensuring community-wide adherence to recycling practices. It establishes clear guidelines and consequences for non-compliance, thereby driving whole communities to actively improve their FW recycling rates. This approach aligns with Taiwan's broader goal of transitioning toward a Zero Waste Society, where waste generation is minimized, and resources are utilized efficiently and sustainably [[70], [71], [72], [73]]. Taiwan's approach to FW management combines financial incentives through recycling funds with strict regulatory measures to ensure compliance with recycling guidelines. This comprehensive strategy not only promotes responsible waste management but also contributes to the overarching vision of a Zero Waste Society, where waste reduction and recycling are integral components of a sustainable future [73].

Through a collaborative effort between the Taiwanese Environmental Protection Agency (EPA) and local governments, an impressive 2100 tons of food waste (FW) are successfully recycled each day in Taiwan [[70], [71], [72], [73]]. This substantial recycling effort yields significant economic benefits, generating approximately 2.7 billion New Taiwan dollars annually [73]. Nevertheless, the program's influence goes beyond financial benefits. Its most notable accomplishment lies in its capacity to enhance consciousness and encourage FW recycling efforts across the entirety of the Taiwanese population. Remarkably, Taiwan's FW recycling initiative has earned accolades and honors, including the National Sustainable Development Award, in acknowledgment of its exceptional role in elevating the recycling rate and guiding Taiwan towards the goal of a waste-free society. This accolade highlights the program's success not only in managing FW effectively but also in aligning with broader sustainability goals. Furthermore, Taiwan's experiences in FW management have garnered international praise and serve as an exemplary lesson for other nations. These practices and lessons have been exported and shared internationally, underscoring their relevance and applicability in diverse contexts [73]. In light of the challenges faced by developing countries in FW management, Taiwan's experiences provide valuable insights. The Taiwanese model, with its emphasis on comprehensive recycling, economic benefits, community engagement, and alignment with sustainable development goals, offers a practical and adaptable approach for addressing FW challenges in developing nations. As such, Taiwan's achievements in FW recycling can serve as an instructive and relevant method for other countries striving to improve their FW management practices [72].

### 5.2 Current Situation of waste management in Malaysia

**Fig. 7:**
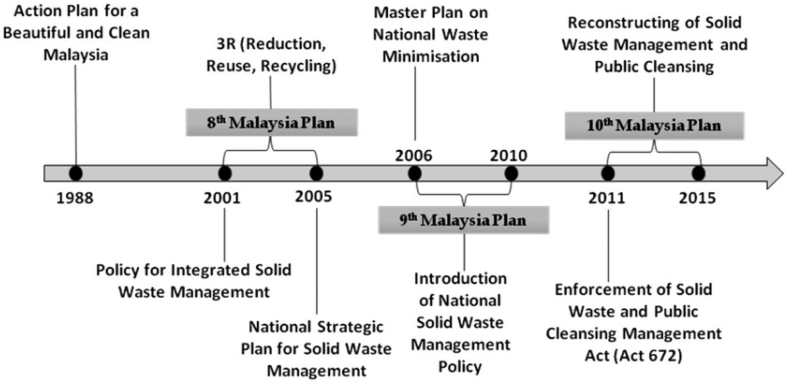
Current situation of waste management in Malaysia [75].

Fig. 7 shows that the current situation of waste management in Malaysia presents several challenges stemming from rapid urbanization and population growth [74]. Landfills are the predominant method of waste disposal, leading to issues such as overburdened sites, environmental concerns, and limited space for waste disposal. Recycling rates, although promoted by the government, remain relatively low, posing ongoing challenges. Furthermore, managing electronic waste (e-waste) and addressing illegal dumping are growing concerns, prompting the government to introduce regulations and enforcement measures [75]. Efforts are also underway to tackle plastic waste issues, including restrictions on plastic imports and the promotion of alternatives to single-use plastics. To gain a comprehensive understanding of the waste management landscape in Malaysia, it is recommended to consult recent reports and official sources for the latest data, policies, and initiatives [76]. Developing a comprehensive food waste management framework is still a work in progress in Malaysia, with many of these frameworks in the planning and development phases [77]. To address the pressing issue of food waste generation, Malaysia could consider adopting successful models and strategies from countries like Japan, Thailand, and South Korea, rather than revisiting the detailed discussion on Taiwan from section 4.1 [74].

For instance, Taiwan's success in establishing an integrative food waste management system could serve as a valuable blueprint for Malaysia. Their rigorous enforcement efforts combined with comprehensive technologies have led to significant reductions in food waste [74]. Taiwan's 'National Development Plan-Green Industry-Resource Recycling and Reuse Program' actively engaged industrial and private sectors in reducing food waste [73]. Thailand has also integrated food waste management policies into its National 3R (Reduce, Reuse, Recycle) strategy, setting ambitious targets for food waste reduction [78]. Japan's 'Food Recycling Law' mandates recycling of food waste into useful products, while South Korea prohibits food waste landfilling and employs a volume-based fee system to manage food waste [79,80]. Despite the progress made by various countries, common challenges persist, including incomplete legislative frameworks, weak enforcement mechanisms, and insufficient budget allocation for food waste management [[76], [77], [78], [79], [80]]. Given the expected intensification of the food waste issue over time, it is imperative for countries like Malaysia to prioritize the establishment of concrete food waste strategies and learn from successful international models to mitigate this pressing challenge. The Solid Waste Management (SWM) Act in Malaysia holds promise for advancing sustainable waste management practices but requires complementary regulations and enforcement to translate policy intentions into effective waste management initiatives [74,75].

### 5.3 Current Situation of waste management in Bangladesh

Managing food waste in Bangladesh presents a multifaceted landscape with several key insights. As the nation experiences rapid urbanization and evolving lifestyles, there is a notable increase in food consumption and waste generation, particularly in urban areas where infrastructure and space are limited [81]. The informal sector, including street vendors and waste pickers, plays a significant role in FW management but requires formalization and support for enhanced efficiency and safety. Awareness about the environmental and economic consequences of FW remains low, emphasizing the need for comprehensive public education [82]. Given resource scarcity challenges, reducing FW can contribute to resource conservation and mitigate economic losses. Addressing FW throughout the food supply chain, strengthening regulatory frameworks, and scaling food recovery initiatives are pivotal steps. Embracing innovation and technology, particularly in cold storage and mobile apps, can further improve FW management. Furthermore, FW management in Bangladesh intersects with climate resilience, making it a critical component of the nation's broader sustainability efforts, necessitating cross-sector collaboration among government, industry, civil society, and consumers. In 2021, food waste management in Bangladesh was confronting a series of complex challenges [83]. The nation was facing a significant increase in food waste generation, particularly in urban areas, driven by rapid urbanization and changing consumption patterns. The informal sector played a crucial role in managing food waste but often lacked formal recognition and support. Efforts were underway to raise awareness about the impact of food waste and promote reduction measures. Bangladesh was also developing policies and regulations to tackle this issue, although implementation remained challenging [82,83]. Given resource scarcity and vulnerability to climate change, efficient food waste management was becoming increasingly vital. Initiatives for food recovery and innovative technologies were emerging, emphasizing the need for cross-sector collaboration to develop comprehensive approaches tailored to the context of developing countries. Applying projects similar to those in developed countries might require adaptation to suit local conditions and capacities.

**Fig. 8:**
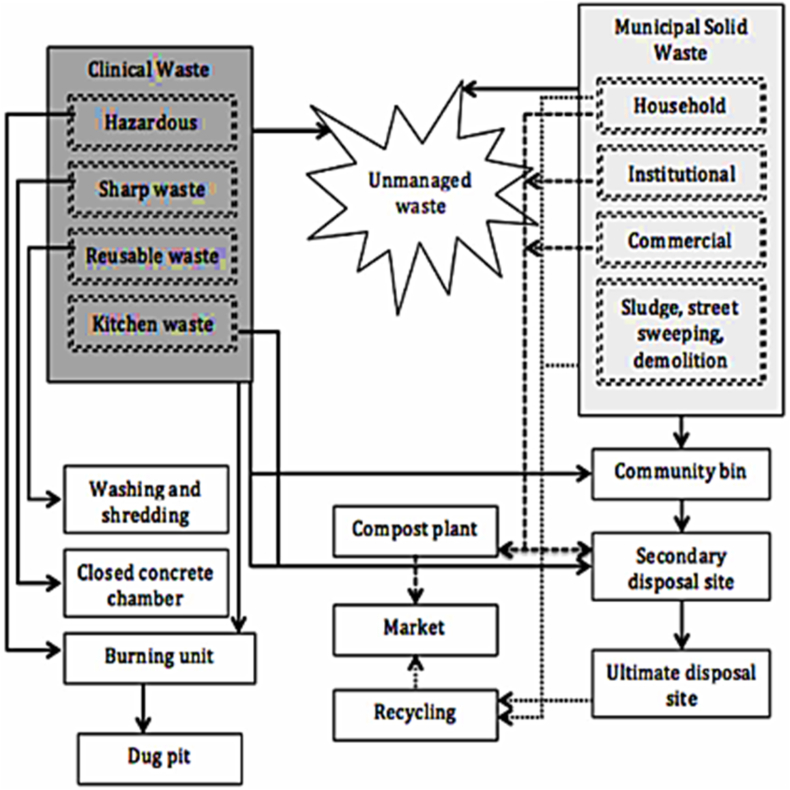
Current situation of waste management in Bangladesh [86].

However, the situation may have evolved since then, necessitating a closer look at the current status of food waste management in Bangladesh as shown in Fig. 8. In urban areas, a concerning trend has emerged with regards to solid waste management. On average, a staggering 55 % of solid waste remains uncollected, leaving a substantial portion of waste improperly managed or disposed. This statistic is based on a study conducted by Ahmed in 2019, highlighting the pressing issue of inefficient waste collection and disposal practices in urban settings [83]. The 55 % of uncollected solid waste signifies a significant gap in the waste management system, where over half of the generated waste is left unattended. This can lead to a range of adverse consequences, including environmental pollution, health hazards, and aesthetic degradation, which affect both residents and the urban environment. Furthermore, the study conducted by Ahmed et al. [83], reveals a considerable variation in collection efficiency, ranging from 37 % to 77 %. This variation indicates disparities in waste management practices across different urban areas. While some regions are more effective in collecting and disposing of waste, others struggle to maintain efficient systems. Understanding these variations is crucial for identifying areas that require immediate attention and improvement in waste collection and management infrastructure. Addressing this issue is of paramount importance. Efficient solid waste management is vital for maintaining the cleanliness and health of urban areas. It reduces the risk of diseases, minimizes environmental degradation, and contributes to the overall well-being of residents [84]. To improve collection efficiency and reduce the percentage of uncollected waste, cities and municipalities should consider investing in modern waste collection systems, public awareness campaigns, recycling initiatives, and stringent waste disposal regulations. The revelation that an average of 55 % of solid waste remains uncollected in urban areas, with varying collection efficiencies, underscores the critical need for improved waste management practices. It is imperative that local authorities, policymakers, and communities collaborate to address this issue comprehensively. By doing so, urban areas can create cleaner, healthier, and more sustainable living environments for their residents [83,84].

In Bangladesh, the 3R policy for waste management plays a pivotal role in addressing the country's growing waste-related challenges. This policy, centered on "Reduce, Reuse, and Recycle," encompasses a multifaceted approach to tackle waste at its source [84]. It encourages industries, businesses, and individuals to reduce waste generation by opting for sustainable alternatives and adopting eco-friendly packaging practices. Additionally, promoting the reuse of items through repairing, refurbishing, and sharing initiatives is actively encouraged. Recycling, a fundamental component of the policy, involves collecting and processing various materials to create new products [86]. Bangladesh's commitment to the 3R policy not only reduces the environmental impact of waste disposal but also conserves natural resources, enhances resource efficiency, promotes public health, and aligns with global sustainability goals [83]. This underscores the nation's dedication to responsible environmental stewardship and a more sustainable future.

## 6 FW management practices between Malaysia and Bangladesh

A comparative analysis of FW management practices between Malaysia and Bangladesh highlights diverse approaches and challenges as shown in Table 5. Malaysia's structured regulatory frameworks, organized waste collection systems, active recycling initiatives, technology adoption, and community engagement provide valuable insights and models that Bangladesh can leverage to strengthen its FW management practices [72]. Conversely, Bangladesh's strong community involvement, informal waste collection networks, and localized solutions offer lessons for Malaysia to enhance community engagement and efficiency in waste reduction and recycling efforts [81]. Through collaboration and knowledge exchange, both countries can advance towards more effective and sustainable FW management systems tailored to their unique contexts and challenges [75]. Food waste management practices in Malaysia and Bangladesh exhibit notable differences shaped by distinct regulatory frameworks, waste collection systems, recycling initiatives, technology adoption, and community engagement approaches [83]. Malaysia's structured regulatory environment and organized waste collection systems offer valuable insights for Bangladesh to enhance its food waste management infrastructure. By adopting Malaysia's successful recycling models and leveraging advanced technologies, Bangladesh can improve efficiency and sustainability in handling food waste [83]. Additionally, Bangladesh's strong community engagement in waste reduction provides an opportunity for Malaysia to enhance its own community involvement strategies [80]. Through mutual learning and collaboration, both countries can benefit from sharing expertise, best practices, and innovative solutions to address the common challenge of food waste mismanagement, ultimately contributing to more effective and sustainable waste management practices in the region [82] (see Table 6).

**Table 5:** Comparative study of food waste (FW) management practices between Malaysia and Bangladesh.

Aspect of FW Management	Malaysia	Bangladesh	Potential Benefits from Each Other
**Regulatory Framework**	Has specific laws and regulations on waste	Regulations primarily address solid waste	Malaysia can share expertise in developing comprehensive FW regulations. Bangladesh can adapt regulatory models from Malaysia.
Waste Collection Systems	Relatively organized with municipal collection	Informal waste collection systems predominate	Malaysia can learn from Bangladesh's informal systems for community engagement. Bangladesh can benefit from Malaysia's structured collection methods.
Recycling Initiatives	Active recycling programs and facilities	Limited formal recycling infrastructure	Malaysia can share knowledge on setting up effective recycling programs. Bangladesh can adopt Malaysia's successful recycling models.
Technology Implementation	Increasing use of technology in waste management	Reliance on traditional waste disposal methods	Malaysia can introduce advanced technologies to Bangladesh for FW management efficiency. Bangladesh can explore technology integration from Malaysia.
Community Engagement	Moderate community involvement in waste reduction	Strong community practices in waste recycling	Malaysia can learn from Bangladesh's community-driven waste reduction initiatives. Bangladesh can benefit from Malaysia's strategies to enhance community participation.

**Table 6:** Food waste management in Taiwan, Malaysia, and Bangladesh.

Parameter	Taiwan	Malaysia	Bangladesh	Reference
**FW Generation Rate (kg/capita/year)**	40	55	85	[95]
**Regulatory Framework**	Strict regulations and enforcement	Moderate regulations, varying enforcement	Limited regulations, weak enforcement	[17,77,95]
**Recycling Technologies**	Advanced (high adoption)	Intermediate (moderate adoption)	Basic (low adoption)	[18,45,95]
**Community Participation Level**	High	Moderate	Low	[66,77,95]
**FW Reduction Initiatives**	Comprehensive national programs	Regional programs, varying effectiveness	Few initiatives, low effectiveness	[95]
**Income Level**	High (GDP per capita: $33,000)	Upper-middle (GDP per capita: $11,000)	Low (GDP per capita: $2000)	[96]
**Population Growth Rate (%)**	0.2 %	1.3 %	1.1 %	[97]
**Technology Diffusion Index**	85 %	60 %	30 %	[95,96]
**Regulatory Compliance Rate**	90 %	70 %	40 %	[95,97]
**Environmental Impact Awareness**	High	Moderate	Low	[95]

Malaysia possesses valuable expertise in regulatory frameworks and advanced waste management technologies that could benefit Bangladesh significantly [73]. Malaysia has established specific laws and regulations governing waste management, including provisions for food waste, which could serve as a model for Bangladesh to develop comprehensive guidelines for food waste management [70]. Additionally, Malaysia has been actively adopting and implementing advanced technologies in waste management, such as smart bins, waste tracking systems, and recycling technologies. By sharing its experiences and best practices in regulatory enforcement and technology adoption, Malaysia can assist Bangladesh in enhancing its waste management practices [74]. This collaboration would contribute to improving efficiency, sustainability, and environmental outcomes in Bangladesh's waste management sector, aligning with both countries' goals for sustainable development and resource conservation. Bangladesh can provide insights into effective community engagement and informal waste collection systems that Malaysia can adopt [79]. Both countries can collaborate to improve their respective FW management practices by exchanging knowledge and implementing successful strategies from each other's experiences.

## 7 Discussion and findings

**Fig. 9:**
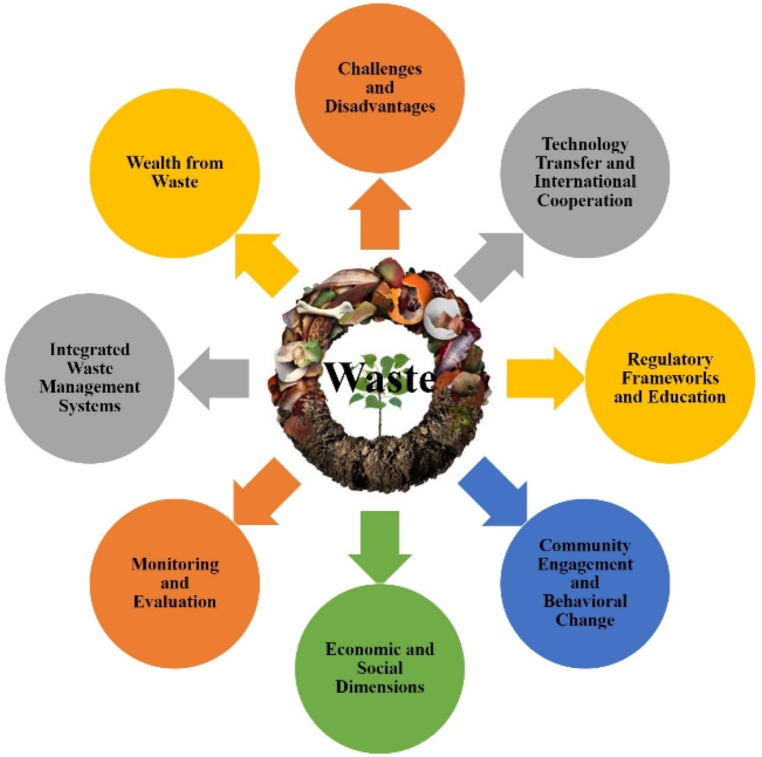
Potential remedies of waste management.

The problem of managing food waste in developing countries is complex and diverse. Based on the existing state of food waste management in these places, our research has highlighted a number of important results and addressed potential remedies as shown in Fig. 9. Ali et al. [87], described that inadequate food waste management systems and legislative measures in developing countries are of concern. Challenges include limited infrastructure, financial and human resource constraints, informal waste picking, inadequate regulations, low public awareness, food security issues, and limited access to advanced waste management technologies. These challenges pose serious health and environmental risks, hinder sustainable development, and exacerbate broader issues like food security and poverty. Craiu et al. [88], reported that the transfer of relevant technologies from developed nations offers promise in addressing food waste challenges. The adoption of advanced methods in countries like China and Thailand signifies the potential for technology-driven solutions. International cooperation plays a vital role in supporting these efforts, facilitating knowledge transfer, and providing valuable shortcuts for developing nations. Shekdar [89], described that the establishing specific regulatory frameworks for food waste management is crucial, as is consistent enforcement and raising public awareness about responsible consumption. Effective enforcement mechanisms are equally vital to translate policies into practical implementation. Pollock et al. [90], stated that the community-based initiatives and private-sector engagement can be pivotal in implementing waste management strategies. Public awareness campaigns and behavioral change initiatives, seen in developed countries, have proven effective in reducing waste generation. Danthurebandara et al. [91], informed that the food waste management is both an environmental and socio-economic concern. Surplus food redistribution to vulnerable populations can address waste reduction and food security issues. Partnerships between governments, NGOs, and the private sector play a vital role in implementing these remedies. Nah et al. [92], reported that the establishing monitoring and evaluation mechanisms is essential to track progress, enabling necessary adjustments to strategies and resource allocation. Batista et al. [93], clarified that developing countries should consider integrated waste management systems encompassing various facets of waste handling, from collection and recycling to energy generation and sustainable practices.

Adetunji et al. [94], designated that the concept of "Wealth from Waste" underscores the potential benefits of waste recycling activities such as composting and animal feeding. In agriculture-based economies, bio-fertilizers produced from waste can meet fertilizer demand sustainably, while using waste as animal feed enhances agricultural productivity and food security in livestock-dependent nations. The research underscores the critical need for developing countries to prioritize and enhance their food waste management systems. By addressing the complex challenges posed by FW generation and disposal, these nations can make significant strides in mitigating food waste's adverse environmental, economic, and social impacts. Developing holistic and integrated FW management frameworks, embracing technology transfer and international cooperation, and fostering public awareness and behavioral change are essential steps toward a more sustainable future.

## 8 Comparison study

The comparative analysis of food waste (FW) management in Taiwan, Malaysia, and Bangladesh reveals significant variations influenced by income levels, regulatory frameworks, technological adoption, and community engagement [95]. Taiwan, with a stringent regulatory framework and advanced recycling technologies, generates the least FW at 40 kg per capita per year, demonstrating high regulatory compliance and community participation [95].

In contrast, Malaysia, with moderate regulations and intermediate technology adoption, produces 55 kg of FW per capita annually, reflecting varying levels of enforcement and community involvement. Bangladesh faces substantial challenges, generating 85 kg of FW per capita per year due to limited regulations, low technology adoption, and minimal community participation. These findings underscore the critical role of comprehensive regulatory measures, advanced technologies, and active community engagement in effective FW management. The study highlights the need for tailored strategies in developing nations, emphasizing the importance of integrated approaches to mitigate environmental and health risks associated with FW mismanagement [[95], [96], [97]].

## 9 Future perspectives

Developing a holistic FW management system shows great potential for promoting sustainable progress in less developed nations. As outlined in research conducted by Xu and colleagues [60], food waste can be efficiently redirected to generate heat or electricity using a method referred to as anaerobic digestion. This approach addresses energy shortages and offers a sustainable solution. Remarkably, one ton of FW has the potential to generate approximately 247 cubic meters of methane gas, equivalent to about 89.78 GJ (GJ) of heating potential or an electrical output of approximately 847 kW-electric (kWe). This demonstrates the feasibility of converting FW into a valuable energy resource [82]. Promoting FW recycling activities can significantly contribute to greenhouse gas emission reduction in developing countries. Research, such as that by Deb et al. [56], has shown that one ton of household FW can generate a carbon impact equivalent to over 3.8 tons of carbon dioxide equivalent emissions. This highlights the importance of FW management efforts in mitigating climate change and controlling environmental pollutants. As countries strive to combat climate change, effective FW management becomes a crucial component of their sustainability efforts [83].

The term 'Wealth from Waste' aptly describes the potential of FW recycling activities, particularly animal feeding and composting. In nations with agriculture based financial prudence, such as Vietnam, India, Thailand, and Indonesia there is high demand for composts. Utilizing FW to produce bio-fertilizers offers an ideal solution to meet this demand sustainably. Additionally, for countries with substantial livestock populations like China, India, and Mexico, using FW as animal feed represents a suitable method that not only reduces waste but also enhances agricultural productivity and food security [84]. Establishing a comprehensive FW management system in developing countries can unlock various opportunities for sustainable development. These include energy generation, greenhouse gas mitigation, and the creation of valuable products like bio-fertilizers, all of which contribute to environmental protection, energy security, and economic growth. Embracing FW management as an integral part of sustainable development efforts is essential for addressing the unique challenges and opportunities faced by developing nations [85].

In an ideal scenario of collaborative food waste management between Malaysia and Bangladesh, Malaysia's expertise in regulatory frameworks and advanced waste management technologies would play a crucial role. Malaysia could share its knowledge by providing guidance on developing comprehensive regulatory frameworks tailored to Bangladesh's needs, ensuring effective oversight and compliance in food waste management. Additionally, Malaysia could assist Bangladesh in setting up organized waste collection systems and promoting recycling initiatives, drawing from its experience in sustainable waste management practices. Bangladesh, in turn, would actively engage in adopting and implementing these strategies, leveraging its strong community networks to drive local participation in waste reduction and recycling programs. Through this collaborative effort, both countries can work towards a sustainable food waste management system that optimizes resource use, minimizes environmental impact, and empowers communities to take ownership of waste reduction efforts. This partnership exemplifies the benefits of international cooperation in addressing common challenges and advancing sustainable development goals.

## 10 Conclusions

This review article highlights a critical issue in developing countries: the inadequacy of comprehensive FW management systems and legislative measures. It underscores the pressing need for effective solutions to address this challenge. To this end, the case study of Taiwan is put forth as an adaptable and instructive model for developing nations seeking to enhance their FW management practices. The review further advocates for the establishment of integrated FW management systems as a strategic approach to guide governments in developing holistic and well-designed FW management frameworks. Such systems would encompass various facets of FW handling, from collection and recycling to energy generation and sustainable practices. Ultimately, the implementation of FW management systems in developing countries holds significant promise. It not only addresses the immediate concern of FW disposal but also presents broader opportunities. These opportunities include meeting energy demands through FW-derived resources, contributing to sustainable development, and fostering environmental stewardship. The review article emphasizes the need for comprehensive FW management solutions in developing countries and recommends the Taiwan case study as a valuable reference. It also underscores the importance of integrated FW management systems in guiding governmental efforts and highlights the positive impact that effective FW management can have on energy sustainability and overall sustainable development in these regions.

## CRediT authorship contribution statement

**Tawfikur Rahman:** Writing – review & editing, Writing – original draft, Validation, Resources, Methodology, Investigation, Formal analysis, Data curation, Conceptualization. **Nibedita deb:** Writing – review & editing, Writing – original draft, Visualization, Methodology, Formal analysis, Data curation. **Md Zahangir Alam:** Supervision, Project administration, Investigation, Funding acquisition. **Md Moniruzzaman:** Writing – review & editing, Visualization, Software, Project administration, Investigation. **Md Shohidullah Miah:** Supervision, Resources, Project administration, Investigation, Funding acquisition. **Mohammad Abu Horaira:** Writing – original draft, Resources, Data curation. **Reashad Kamal:** Resources, Data curation.

## Declaration of competing interest

The authors declare that they have no known competing financial interests or personal relationships that could have appeared to influence the work reported in this paper.
